# Gap-enhanced Raman tags: fabrication, optical properties, and theranostic applications

**DOI:** 10.7150/thno.39968

**Published:** 2020-01-12

**Authors:** Nikolai G. Khlebtsov, Li Lin, Boris N. Khlebtsov, Jian Ye

**Affiliations:** 1 Institute of Biochemistry and Physiology of Plants and Microorganisms, Russian Academy of Sciences, 13 Prospekt Entuziastov, Saratov 410049, Russia; 2 Saratov National Research State University, 83 Ulitsa Astrakhanskaya, Saratov 410026, Russia; 3 State Key Laboratory of Oncogenes and Related Genes, School of Biomedical Engineering, Shanghai Jiao Tong University, Shanghai 200030, P. R. China; 4 Department of Nuclear Medicine, Ruijin Hospital, School of Medicine, Shanghai Jiao Tong University, Shanghai 200025, P.R. China; 5 Shanghai Key Laboratory of Gynecologic Oncology, Ren Ji Hospital, School of Medicine, Shanghai Jiao Tong University, Shanghai 200127, P. R. China

**Keywords:** surface-enhanced Raman scattering (SERS), gap-enhanced Raman tags (GERTS), plasmonic core-shell nanoparticles, bioimaging, plasmonic photothermal therapy, theranostics

## Abstract

Gap-enhanced Raman tags (GERTs) are emerging probes of surface-enhanced Raman scattering (SERS) spectroscopy that have found promising analytical, bioimaging, and theranostic applications. Because of their internal location, Raman reporter molecules are protected from unwanted external environments and particle aggregation and demonstrate superior SERS responses owing to the strongly enhanced electromagnetic fields in the gaps between metal core-shell structures. In this review, we discuss recent progress in the synthesis, simulation, and experimental studies of the optical properties and biomedical applications of novel spherically symmetrical and anisotropic GERTs fabricated with common plasmonic metals—gold (Au) and silver (Ag). Our discussion is focused on the design and synthetic strategies that ensure the optimal parameters and highest enhancement factors of GERTs for sensing and theranostics. In particular, we consider various core-shell structures with build-in nanogaps to explain why they would benefit the plasmonic GERTs as a superior SERS tag and how this would help future research in clinical analytics and therapeutics.

## 1. Introduction

Plasmonic enhancement of local electromagnetic (EM) fields by metal nanostructures is the key physical process behind surface-enhanced Raman scattering (SERS) spectroscopy. Raman spectra provide unique information about molecular vibration modes, thus ensuring analytical means for the detection of molecules and their local environment in condensed phases. Unfortunately, the Raman scattering cross-section is too small (10^-30^-10^-24^ cm^2^/sr) to be used in practice in the same way as, for example, the typical scattering cross-sections of fluorescent dyes (about 10^-16^ cm^2^/sr) 1. That is why Raman spectroscopy found wide application only after the EM nature of SERS enhancement was discovered and explained 2-4. At present, owing to the significant progress in the SERS devices available on the market and because of the spectacular experimental demonstrations of the SERS possibilities in analytical, bioimaging, and theranostic applications, this technique has become a widespread laboratory tool. Nevertheless, the investigation of various SERS tags and substrates is still a topical problem of practical importance.

There are several ways to enhance the local EM fields through the EM coupling of excited modes, including the formation of a small nanogap between plasmonic particles or between particles and tips or flat metal surfaces. Although such nanostructures can be fabricated through modern controllable technologies with predicted geometrical parameters, the SERS response may suffer from possible unwanted interference effects of the surrounding media. From this point of view, nanostructures with Raman molecules (RMs) embedded into internal nanogaps seem very promising for biomedical applications. Since the pioneering publication of Nam's group 5, SERS core/shell tags with inner nanogaps (also called gap-enhanced Raman tags—GERTs 6) have attracted considerable attention because of their following advantages: (1) RMs are protected from desorption and environmental conditions; (2) they are subjected to a uniform and strongly enhanced EM field in the gap; (3) GERTs produce a stable SERS response even if aggregated; (4) the highly uniform spectral pattern ensures a linear correlation between probe concentration and SERS intensity; and (5) GERT probes can be multiplexed by incorporating different RMs into two-layered or multilayered GERTs.

The SERS topics have been extensively reviewed during the past years. Among the many available reviews, most detailed considerations can be found in Refs. 7-11. However, only one review, by Nam *et al*. 12, was specially focused on the nanogap-enhanced Raman tags. Given that there have been many publications on the fabrication and biomedical application of GERTs in recent years, we aimed to provide updated information on their synthesis, optical properties, and theranostic applications. As distinct from the above-mentioned review by Nam *et al*. 12, our consideration includes recent data (2015-2019) on the synthesis and plasmonic properties of GERTs and on biomedical and theranostic topics such as analytical sensing, *in vivo* and *in vitro* bioimaging, and cancer intraoperative theranostics.

## 2. Synthesis of plasmonic nanoparticles with embedded Raman reporters

### 2.1 General scheme of GERT synthesis

**Figure 1:**
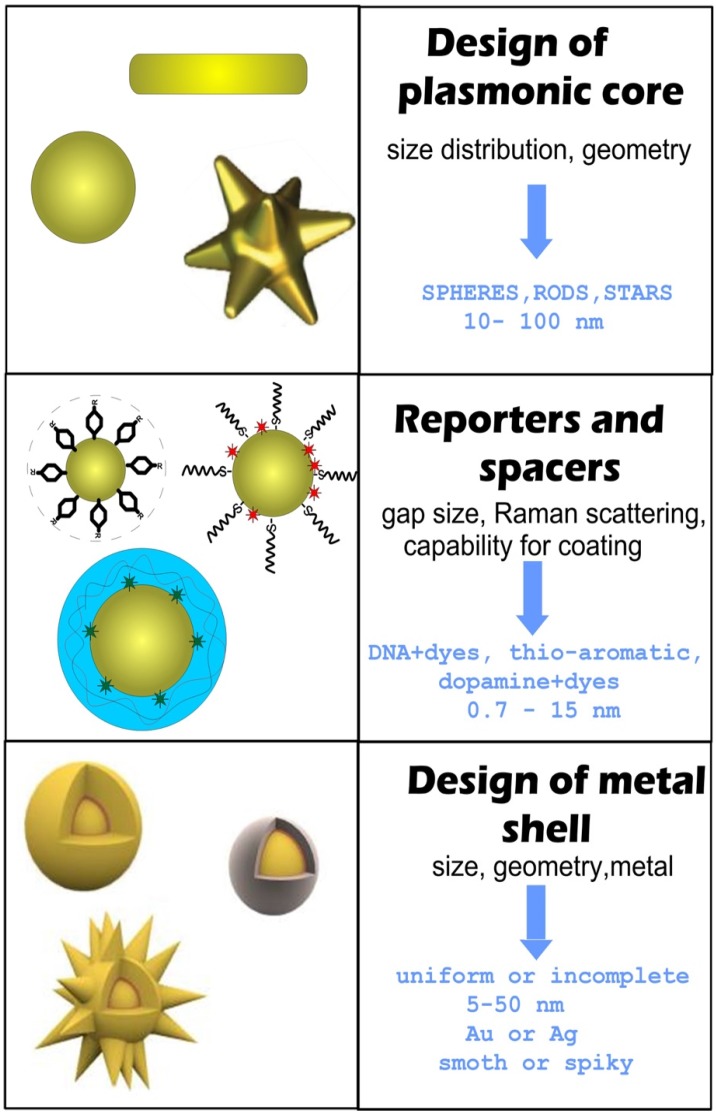
General steps and design criteria in engineering of reporter-embedded gap-enhanced Raman tags (GERTs).

For RMs embedded in the nanogaps inside core/shell nanoparticles (NPs), the SERS intensity is largely affected by the design of the plasmonic nanostructures and by the location of reporters in the hot spots 12, 13. In this context, the design and synthesis of nanogap structures with high precision, yield, and position control for Raman active molecules are key requirements for the successful fabrication of effective GERTs. Protocols for the synthesis of gap-based SERS platforms can be implemented by various technologies, including electron beam lithography 14, 15 and atomic layer deposition 16, 17. However, for colloidal SERS tags with embedded reporters, a typical wet chemical approach consists of three main stages (Figure 1).

The first stage is the design and synthesis of a plasmonic core. The important points here are the initial size of NPs, their monodispersity, and their capacity for robust conjugation with Raman reporters and spacers. The functionalization of the cores with reporters and spacers is a key step that determines the structure of the gap, the position of the reporter molecules inside the particle, and therefore the final enhancement of the SERS. Generally, when thiolated aromatic molecules 18, Raman active polymers 19, or labeled oligonucleotides 5 are used as the embedded Raman reporters, they can also serve as spacers. An ideal NP functionalization should provide a high concentration of SERS active molecules, be able to adjust the thickness of the spacer layer between 0.7 and 10 nanometers, and be suitable for robust metallization. At the last stage, a secondary shell is formed by the directional reduction of the metal on the functionalized core surface.

There are two main strategies for the directional growth of secondary shells. One is based on the preferential chemical reduction of Au in the between of or on the spacer molecules. At the initial stage, small Au islands are formed near these points, and then the primary islands grow together, forming a complete shell. In this case, the reporter molecules inside the particle are placed in a gap with bridges 20. The other strategy is used when a polymer layer serves as a spacer and the metallization of the polymer surface forms a hollow gap between the core and the shell 19.

In addition to GERTs with hollow and nanobridged gaps, one should differentiate between GERTs with complete and incomplete shells. GERTs with complete shells have an important advantage over those with incomplete shells, because their SERS responses demonstrate low variations between the fabricated particles. As a rule, the shell thickness ranges from 5 to 50 nm, whereas the optimal shell thickness values for the highest SERS signal depend on many parameters, such as the shape of the core, the thickness of the gap, and the shell material. The formation of spiky or petal-like shells can produce a stronger field inside the particle gap (compared to smooth shells) and, accordingly, higher SERS signal values, as compared to smooth shells 21.

### 2.2 Spherical core/shell Au@Au GERTs

#### 2.2.1 GERTs with oligonucleotides as spacers

Existing synthetic protocols allow the fabrication of plasmonic cores of different shapes ranging from simple spherical colloidal Au particles to more complex structures such as nanostars and nanocages. Despite the diversity of available particle types, the spherical particles are most widely used as the core of the SERS tags with embedded reporters. The main reason is the simplicity of making monodisperse particles in a wide range of sizes. In general, NPs obtained by the Frens citrate reduction method 22 (the more appropriate name could be the Borovskaya-Turkevich-Frens method 23) are suitable candidates as plasmonic cores for GERTs. However, the particles obtained by seed-mediated growth in a cetyltrimethylammonium chloride (CTAC) solution have a narrower size distribution and a more spherical shape 24, 25. Furthermore, such particles are stabilized by the CTAC bilayer, which makes them colloidal stable in saline buffers and very convenient for conjugation with thiolated molecules by simple mixing without aggregation.

**Figure 2:**
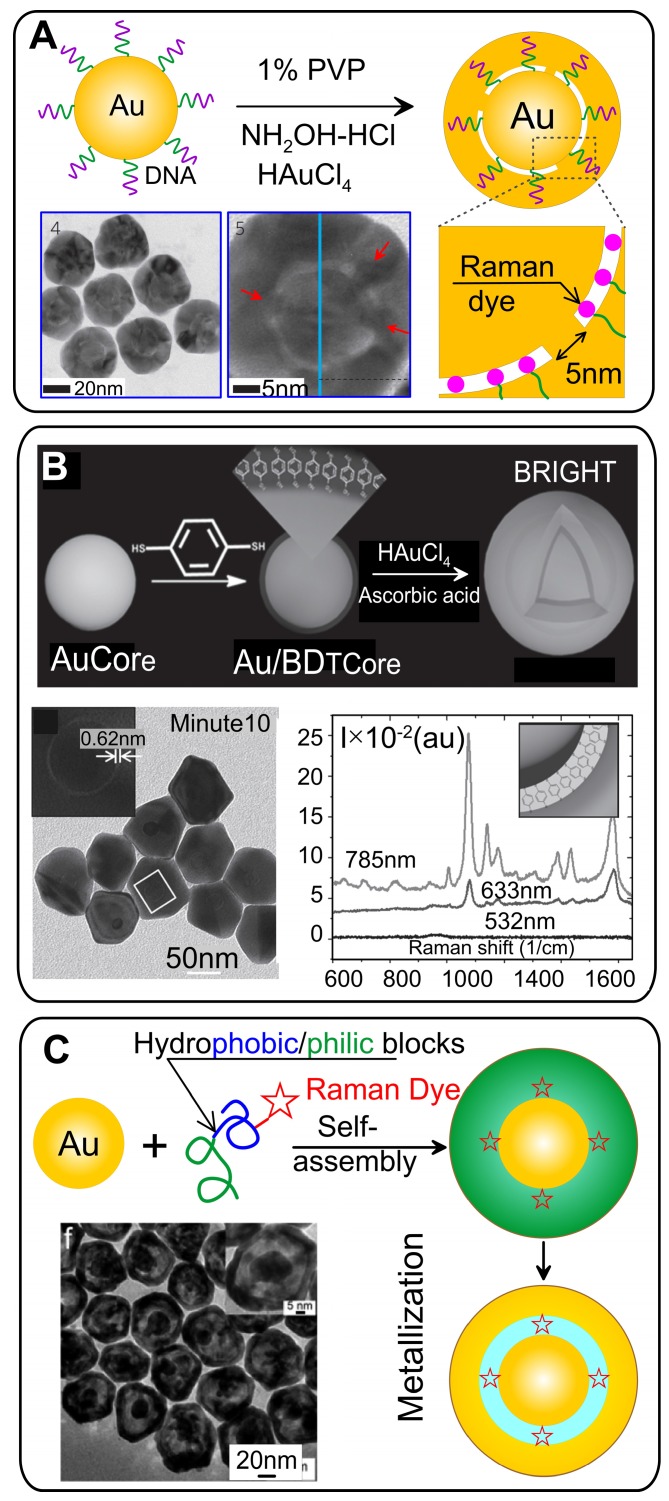
(A) Synthetic scheme for the spherical Au@Au GERTs by using DNA-modified Au NPs as templates. Adapted from Ref. 5. (B) Design and synthesis of Au@Au GERTs by using thiolated aromatic molecules as reporters and spacers. Adapted with permission from Ref. 18. (C) Schematic illustration of the synthesis of nanogapped AuNPs on the basis of self-assembly of amphiphilic block copolymers on the Au core surface. Transmission electron microscopy (TEM) image illustrates the GERT structure with an interior nanogap of 1.5 nm. Adapted with permission from Ref. 19.

The first protocol of reproducible synthesis of SERS probes in which RMs were embedded in a uniform 1 nm gap was developed by Lim *et al*. 5. They used citrate stabilized commercial 12 nm spherical Au particles as cores to modify them with oligonucleotides possessing a Raman dye and a thiol group. The final step involved the formation of a uniform Au shell by the reduction of HAuCl_4_ in the presence of a stabilizer and a mild reduction agent (Figure 2A).

Because the anchored DNA strands facilitate the formation of bridged nanogaps, such nanostructures are revealed by high-resolution transmission electron microscopy (HRTEM; Figure 2A). The precise positioning of Raman dyes inside the gap generates a strong and highly stable SERS signal with enhancement factor (EF) values narrowly distributed in the range of 1.0 ×10^8^ to 2.0 × 10^9^, as measured with a single-particle experiment under 633-nm laser excitation. The extremely high EF values are associated with the strong EM field in the interior nanobridged gaps. EM simulations for wavelengths of 514, 633, and 785 nm showed a strong wavelength dependence of the EFs, in agreement with the experimental data, while with hollow gap structures, no significant incident wavelength dependence was observed. After the publication of this keynote paper, many attempts have been made to understand and improve the DNA-mediated synthesis of GERTs 26, 27.

Obviously, the modification of cores with a DNA spacer is a key point in the control of nanogap morphology and the corresponding SERS properties of the dye molecules inside the gap. To understand how the modification of AuNP surface with thiolated DNA affects the formation of the interior nanogap, the authors varied the DNA base, length, sequence, and grafting density during the synthetic process 28, 29. Oh *et al*. 29 found that different interior nanogap structures were formed when different thiolated DNA sequences were used. In particular, different DNA sequences (poly A, T, G, and C) generated different nanogaps, mainly because of the different binding affinities between the different DNA bases and the AuNP core surface 29.

The differences in NP structure led to differences in the SERS response. For example, Au GERTs prepared with T10 spacers showed the strongest SERS intensity—3.6 times higher than that with the A10 spacer under 633 nm excitation and 7.7 times higher under 785 nm excitation 28. It should be noted that oligonucleotides of different lengths do not precisely control the thickness of the gap inside the particles. For example, the A5, A10, A20, and A30 spacers generated very similar structures with similar 1.2 nm interior nanogaps, and T10 and T30 resulted in very similar GERTs with identical geometrical and SERS properties 29. This is because only a short 10-base DNA fragment directly bound to the Au surface by a thiol bond determines the kinetics of shell growth. On the other hand, the grafting density of nucleotides can be controlled by varying salt-aging conditions 30, thus affecting the morphology of the final particles. The overall trend is that no interior gap is formed when the DNA grafting density is low (about 10 molecules per particle) and a more distinctive interior gap is formed with an intermediate or high DNA grafting density. In the absence of the intragap, the SERS signal from embedded RMs is very weak, and it increases as the DNA grafting density increases to initiate the formation of more bridged gaps. However, for a very high density of adsorbed oligonucleotides, the SERS response can be reduced significantly because of the formation of particles with incomplete secondary shells.

In addition to the type and grafting density of oligonucleotides, another important process affecting the structure of GERTs and SERS properties is the growth kinetics of the Au shell at the third stage of particle synthesis. Lee *et al*. 31 demonstrated the influence of the pH and the ion concentration, on the resulting Au shell structure and on SERS properties. In contrast to variation in oligonucleotide length, in this case the intrananogap distance could be varied from 0.9 nm to 4.0 nm. Depending on the reaction conditions, open- and closed-shell structures were observed, including star-shaped GERTs. Half-shell exhibited a very strong SERS response with an NIR excitation wavelength of 785 nm, whereas closed-shell GERTs with narrow gaps generated intense responses only at short wavelengths (532 nm). GERTs with large gaps were ineffective at all wavelengths tested. Note that these experimental findings contradict the theoretical estimates by the same group 29, which predicted smaller EFs for GERTs with incomplete shells, as compared with complete-shell GERTs at 633-nm excitation.

Finally, the important parameters determining the field enhancement inside Au-NNPs are the thickness and surface roughness of the final GERTs. Hu *et al*. 32 showed that 30 nm Au shells lead to the maximal SERS response from Raman-active molecules embedded into nanogap. Similarly, Lee *et al*. 33 demonstrated that the ratio between reductant/Au^3+^ is key to control the surface roughness. In turn, the roughness of the particle surface greatly affects and enhances the EM field not only in the area of hot spots on the particle surface but also in the intragap, thus resulting in a one or two orders of magnitude higher SERS response.

In summary, for fabrication of DNA-based GERTs with the best properties, it is important to modify the core with the right DNA sequence under optimized salting conditions and with the optimal kinetics of Au shell growth. It should be noted that in all the above-cited works, only citrate-stabilized spherical Au particles (10-20 nm) were used as the GERT cores. This is because only for such particles is it possible to control the oligonucleotide grafting density and, therefore, the kinetics of Au shell growth. Finally, all those papers have reported the use of standard DNA labeled with fluorescent dyes such as Cy3, Cy5, ROX, rhodamine 6G (R6B), or and 4,4-dipiridyl as Raman reporters.

#### 2.2.2 GERTs with thiolated aromatic molecules

The widely used Raman reporters for SERS tags are thiolated aromatic molecules, which possess a number of promising modalities 7. Because of the high chemical affinity of the thiol group to the metal surface, thiolated molecules can easily be conjugated to a wide range of NPs by simple mixing. What is more, Raman spectra with relatively few characteristic peaks are essential for preparing tags for multiplex labeling to avoid signal overlap from the tags. Gandra *et al*. 18 pioneered the use of 1,4-benzenediol (1,4-BDT) molecules as reporters and spacers for synthesizing GERTs, which were also called bilayered AuNP nanostructures with hidden tags. Synthesis of such GERTs starts with the synthesis of CTAC-stabilized spherical Au particles as cores, followed by conjugation with a monolayer of 1,4-BDT (Figure 2B) and growth of Au shells on the cores by mixing the Au salt HAuCl_4_ with a capping agent (CTAC) and ascorbic acid as a mild reducing agent. HRTEM images of the resulting particles reveal a sub-nm gap between core and shell, which indicates the successful trapping of the Raman reporters between the core and the shell. Remarkably, the Raman spectra reveal an 8 cm^-1^ red shift in the CH bending mode and a 20 cm^-1^ red shift in the phenyl ring stretching mode, as compared to 1,4-BDT adsorbed onto a planar SERS substrate because of the compressive stress on 1,4-BDT between the metal layers. The particles had the highest SERS intensity for 785-nm excitation, a nearly fourfold lower intensity for 633 nm, and almost no signal for 532 nm. Note that 1,4-BDT-embedded GERTs demonstrated a SERS response of an order of magnitude higher than that obtained with other particles with surface-adsorbed 1,4-BDT molecules.

We have also demonstrated that not only 1,4-BDT but also other thiolated aromatic molecules, such as 4-aminothiophenol (4-ATP) and 4-methylbenzenethiol (4-MBT), can be used to synthesize core/shell particles with embedded Raman reporters and distinct gaps 34, 35. Lin *et al*. 34 showed that the synthesis strategy can be repeated several times to fabricate multilayered particles, called plasmonic multishell nanomatryoshkas (NMs). Single-shell, double-shell, and triple-shell NMs were synthesized with built-in 1,4-BDT and 4-MBT molecules. The averaged SERS measurements with aqueous samples demonstrated the best performance of double-shell NMs with a ~22-times stronger SERS signal, as compared to their single-shell counterparts. Moreover, the double-shell NMs showed a combined SERS signal when 1,4-BDT and 4-MBT were separately embedded in different gaps, which makes such particles suitable for multiplexing.

The structure and thickness of the molecular layer formed by the reporter molecules inside the particle determine the magnitude and spectral setting of the maximum Raman scattering gain. It can be assumed that the gap size should correspond to the size of the aromatic spacer molecule. The gap-size distribution diagram for Au@BDT@Au NPs 36 showed a most probable peak centered at 0.72 nm, which corresponded to a slightly tilted 1,4-BDT configuration at a low surface coverage; and the second and third peaks were centered at 1.24 and 1.76 nm, which were attributed to a bilayer and a trilayer of tilted 1,4-BDT molecules, respectively. If different derivatives are used as spacers (for example, 1,4-BDT, 4,4′-biphenyldithiol, and 4,4′-terpheyldithiol), the gap thickness depends both on the length and the incubation time of Au cores with spacers 37. The accurate measurement of plasmonic shifts during the adsorption of Raman reporters on the core, together with measurements of layer thickness distribution, can be used to quantify the refractive index of nanometer-thick thiolated molecular layers 38. The obtained values ranged from 1.59 to 1.66 and were relatively higher than the value measured by ellipsometry (1.45).

In addition to the thickness of the molecular layer, the particle structure should have a significant effect on SERS properties such as the presence of Au bridges between the core and the shell. Unlike DNA-mediated gap formation, in most works devoted to the synthesis of gapped particles with embedded thiol-aromatic molecules, only monolayer coverage has been reported without any control of grafting density. By contrast, we demonstrated that more distinct bridged nanogaps were formed inside GERTs through the use of aromatic molecules with only one thiolated end group (in this case, 4-MBT 36). However, the SERS response for 4-MBT-embedded particles was lower than that for 1,4-BDT-embedded GERTs.

**Figure 3:**
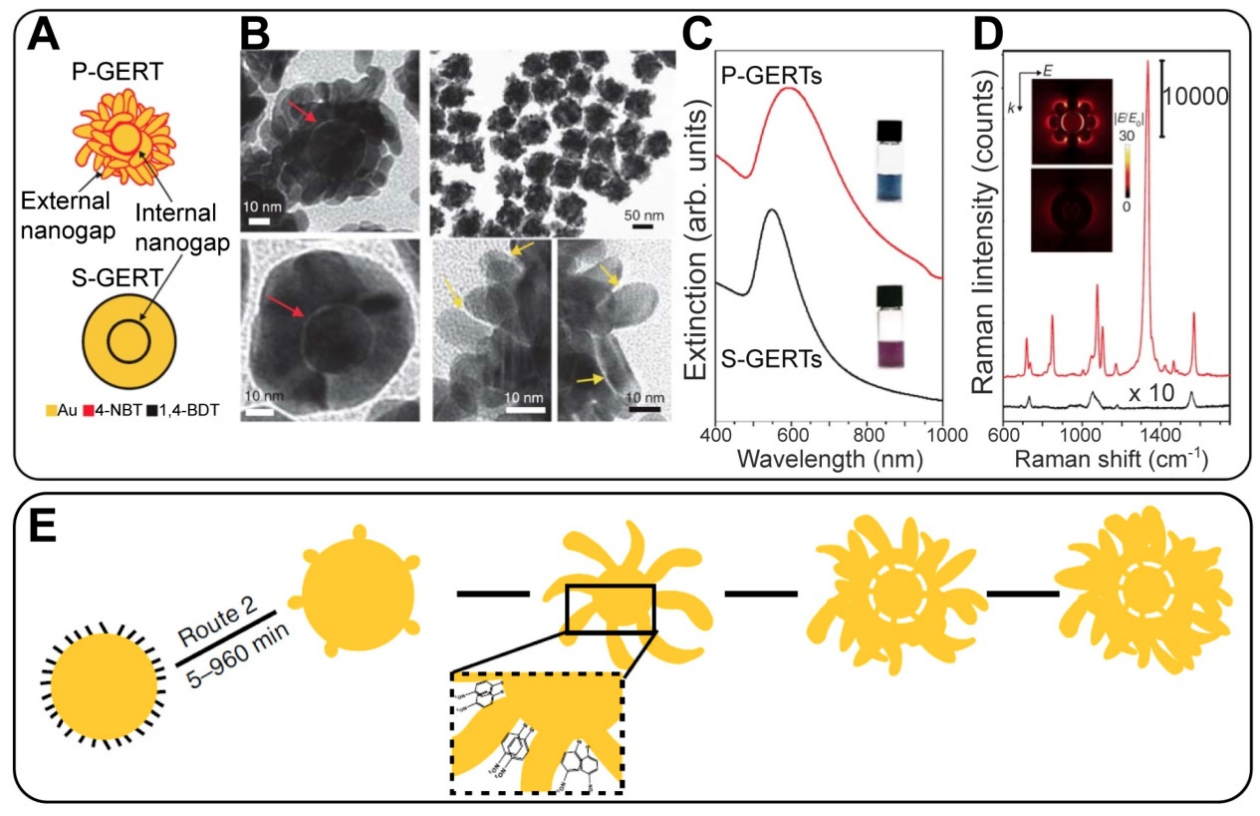
Schematic structures and the corresponding representative TEM images of P-GERTs (A, B) and S-GERTs (C, D); Red and yellow arrows indicate the internal and external nanogaps, respectively. The panels (F) and (G) represent sample photos and extinction spectra, FDTD simulations, and SERS spectra. The bottom panel (H) illustrates the synthetic scheme of P-GERTs. Reproduced with permission from Ref. 39.

GERTs with spherical symmetry and smooth outer shells demonstrate usually high but not extraordinary EFs. For increasing the SERS performance of gap-enhanced tags, several attempts have been made to modify the outer shell morphology in a starlike 21 or an irregular roughness manner 33. Still, there is an urgent need for a strong increase in the GERT efficiency down to the single-particle level. Recently, we fabricated a new version of GERTs with a spherical core, a hollow gap, and a branched petal-like shell structure (for brevity, such particles were called P-GERTs; Figure 3) 39. Because of the generation of strong EM hot spots in both the internal gap and the petal-like structures, the fundamental EF was as high as 5 × 10^9^, thus enabling single-particle detection. Remarkably, the synthetic protocols of both conventional GERTs (S-GERTs) and P-GERTs are very similar and differ mainly in the use of 1,4-BDT or 4-nitrobenzenethiol (4-NBT) as the spacer.

#### 2.2.3 GERTs with Raman-active polymers

Significant disadvantages of the above-discussed nanogapped particles are the inability to adjust the size of the gap in a wide range from 1 nm to tens of nanometers, as well as the restriction on the possible number of reporter molecules in the gap. To overcome these disadvantages, Song *et al*. 19 developed a new strategy to synthesize core-shell metal NPs with an interior, Raman tag-encoded nanogap by taking advantage of the NP-templated self-assembly of amphiphilic block copolymers and localized metal precursor reduction by redox-active polymer brushes. The polymerization of amphiphilic block molecules around the Au core leads to hybrid NPs, with the Raman dye-tagged hydrophobic block sandwiched between the Au core and the hydrophilic polymer brush shell (Figure 2C). The shell can subsequently reduce the Au precursor locally to form an integral Au nanoshell, giving rise to nanogapped NPs with Raman tags positioned inside the nanogap. Further, the same group extended this approach by using dopamine as a precursor for a polydopamine (PDA) shell 40. PDA is an optimal spacer candidate because of a set of useful physicochemical properties: (1) dopamine deposits from aqueous buffer onto any solid substrate, forming a coating with a precisely controlled thickness in the nanometer scale 41, 42; (2) the high density of the functional groups, facilitating in situ nucleation and deposition of a metallic layer; (3) the click reaction of the quinone groups in PDA with thiols and amines makes it possible to encode the nanogaps with various Raman reporters at a high density.

This promising technique produces a SERS-encoded nanogap with a tailorable thickness ranging from 1 nm to tens nanometers. Both the tunable gap and various reporter types are crucial for the optimization of GERTs to the designed laser wavelength and most effective plasmonic properties with respect to the maximal SERS response 13. For example, GERTs with rhodamine B incorporated into a 2-nm gap demonstrate the highest SERS response under 633 nm laser excitation. Importantly, the universal adhesion of dopamine enables the making of NPs with highly tunable geometrical and optical properties, including multiple concentric metallic shells 43, nonspherical nanogapped NPs, and complex magnetic/plasmonic nanostructures 40.

In other examples, silica 44 and polyelectrolytes 21, 45 were utilized as an interlayer to facilitate the formation of nanogap particles with designed thickness. However, the advantages of such approaches, as compared with PDA-based technologies, in terms of gap size tuning and simplicity of synthesis are not clear.

### 2.3 Au@Au probes with nonspherical cores

Spherical AuNPs have two drawbacks, which reduce their effectiveness as the core of GERTs. First, their plasmon resonance is in a narrow spectral range (520-540 nm). For many biomedical applications, it would be preferable to use NPs with a plasmon resonance in the tissue transparency window at 750-900 nm. It should be noted that for GERTs with a spherical core and a relatively thick gap, the extinction spectrum is determined mainly by the secondary shell (in many aspects, similar to the spectrum of SiO_2_/Au nanoshells) and can be tuned to the desired spectral range. However, for such particles, the SERS response is very weak.

The other drawback of spherically symmetrical GERTs is that spherical Au particles themselves produce a moderate EM enhancement and, accordingly, a rather low SERS response. The use of nonspherical cores can be a more promising approach to fabricate GERTs with better SERS performance, as compared to nanosphere-based GERTs. Finally, the third possibility is related to the potential ability of the core surface morphology to control the structure of the gap and the SERS response of NPs 20.

**Figure 4:**
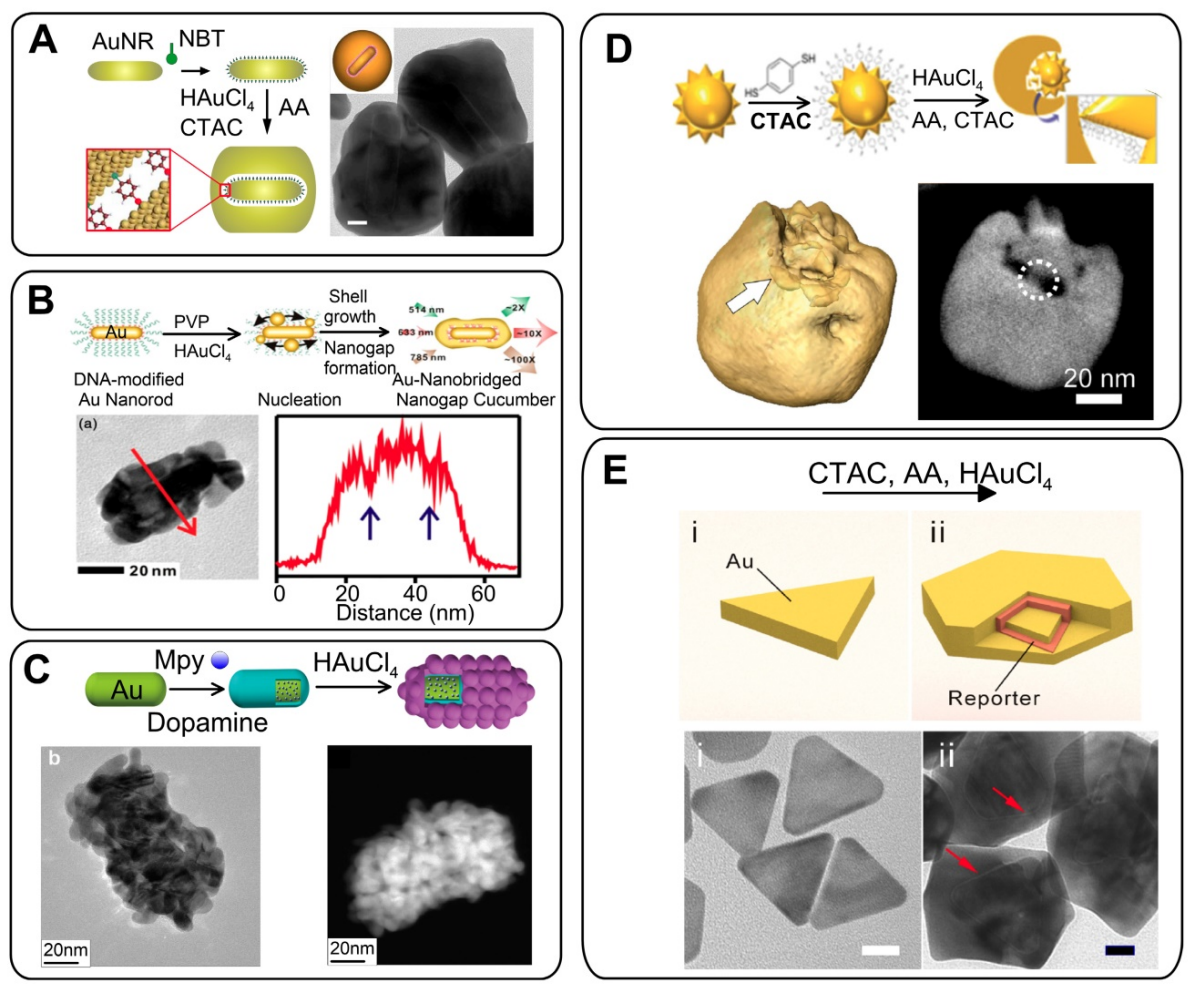
(A) Three steps in GERT synthesis. TEM image of GERTs with a 1-nm gap between AuNR core and shell. Adapted with permission from Refs. 46, 47. (B) Synthetic strategy and TEM image of Au nanocucumbers. Element line mapping analysis of the region is shown by red arrows. The gap cavity regions are indicated by blue arrows. Adapted with permission from Ref. 48 (https://pubs.acs.org/doi/10.1021/acsomega.8b01153, further permissions related to the material excerpted should be directed to the ACS). (C) Schematic synthesis of GERTs based on polydopamine-coated AuNRs. HRTEM and STEM images of GERTs with interior gaps. Adapted from Ref. 49 (D) Schematic illustration of the expected Au-nanostar-seeded growth of GERTs. 3D rendering of an electron tomography reconstruction for a semishell covered Au nanostar. A slice through the reconstruction reveals the connections and gaps between seed and shell. Adapted with permission from Ref. 50. (E) Schematic illustration and TEM images of Au nanotriangles (i) and nanotriangle-based gap-enhanced Raman tags (ii). Adapted with permission from Ref. 53.

The NPs of the first choice with a tunable plasmon resonance, a high SERS response, and relatively low polydispersity are Au nanorods (AuNRs). On the basis of this approach, we developed nonspherical GERTs with RMs embedded in a 1-nm gap between Au nanorod core and Au shell 46. The synthetic protocol was similar to that used for the spherical core tags (Figure 4A). Such GERTs have a strong and uniform SERS response - an order of magnitude higher than that of other common SERS tags such as Au nanorods, nanostars, Au nanoshells with RMs on the surface, and spherical GERTs with embedded reporters.

Our SERS measurements and EM field calculations for AuNR-based GERTs show the generation of strong hot spots that are isolated and protected from the environment by an outer Au shell. This greatly improves the SERS intensity and uniformity, as compared to those observed for AuNRs with surface-adsorbed molecules 47. As we noted above, for AuNR-based GERTs, the synthesis strategies are much the same as for spherical Au cores. For example, Hwang *et al*. 48 reported the synthesis of AuNR-nanogapped particles (so-called Au nanocucumbers, Figure 4B) from DNA-modified Au nanorods and compared their SERS response with that from common nanosphere-based GERTs. The nanocucumbers generate a high SERS response under different laser excitations (514, 633, 785 nm), whereas spherical GERTs have high SERS only under 633-nm excitation. Recently, Zhang *et al*. 49 used AuNRs as a core to develop a novel spiked rodlike core-molecule-shell nanostructure with an interior gap formed by polydopamine. The Raman active molecules were embedded in the gap and produced an internal reference signal, whereas the spiky outer Au surface was used for SERS detection of analytes (Figure 4C).

Other types of plasmonic particles have also been used as cores for GERTs, with the predominant use of thiolated RMs as spacers and reporters. For example, Liz-Marzán and coworkers 50 reported a strategy that combines Au nanostars (AuNSt) as a core and 1,4-BDT as a Raman reporter to produce exotic nanostructures in which internal gaps are revealed through 3D electron tomography (Figure 4D). SERS characterization showed that semishell-coated nanostars display a higher SERS intensity, as compared to other geometries obtained along the seeded growth process. Using the same synthetic strategy, Yang and Jiang 51 showed that the overall Raman scattering signal from AuNSt@BDT@Au nanostructures was 20-30 times stronger than that from AuNP@BDT@Au. Additionally, RMs were adsorbed onto the outer surfaces of AuNS@BDT@Au and exhibited only about 5% of the Raman signals from the embedded molecules. Of note, other types of thiolated RMs (e.g., 5-mercaptobenzene acid 52 can also be used as Raman reporters for AuNSt-based GERTs without any significant decrease in the SERS response. Very recently, we have successfully developed a new Au nanotriangle-based GERT with 1,4-BDT-embedded RMs (Figure 4E). Because nanotriangles have many sharp corners, the fabricated GERTs showed greatly improved SERS enhancement (almost 20-fold greater than that from conventional sphere-based GERTs) and photothermal performance 53.

In general, the number and quality of synthetic protocols for GERTs on nonspherical cores are rather limited, as compared to those of protocols for spherical GERTs. Further study should be focused on the synthesis of GERTs with controllable gap size and on the use of other types of NPs as cores (e.g., nanocubes). However, even at the current stage of research, one can make several important general points related to GERTs on nonspherical cores. First, the SERS response from these particles is typically 20-30 times higher than that from spherical-core-based GERTs. Second, the total size of nonspherical GERTs and their polydispersity are also higher than those of spherical counterparts. Third, the semishell coating produces a higher SERS response than the full coverage. And finally, we have shown that GERTs themselves can serve as cores for the subsequent synthesis of double- and triple-shell Au GERTs, so-called nanomatryoshkas 34. SERS measurements performed with these samples indicate that the double-shell NMs generate a 20 times stronger SERS signal, as compared to that of the usual GERTs.

### 2.4 Au@Ag SERS tags with embedded reporters

**Figure 5:**
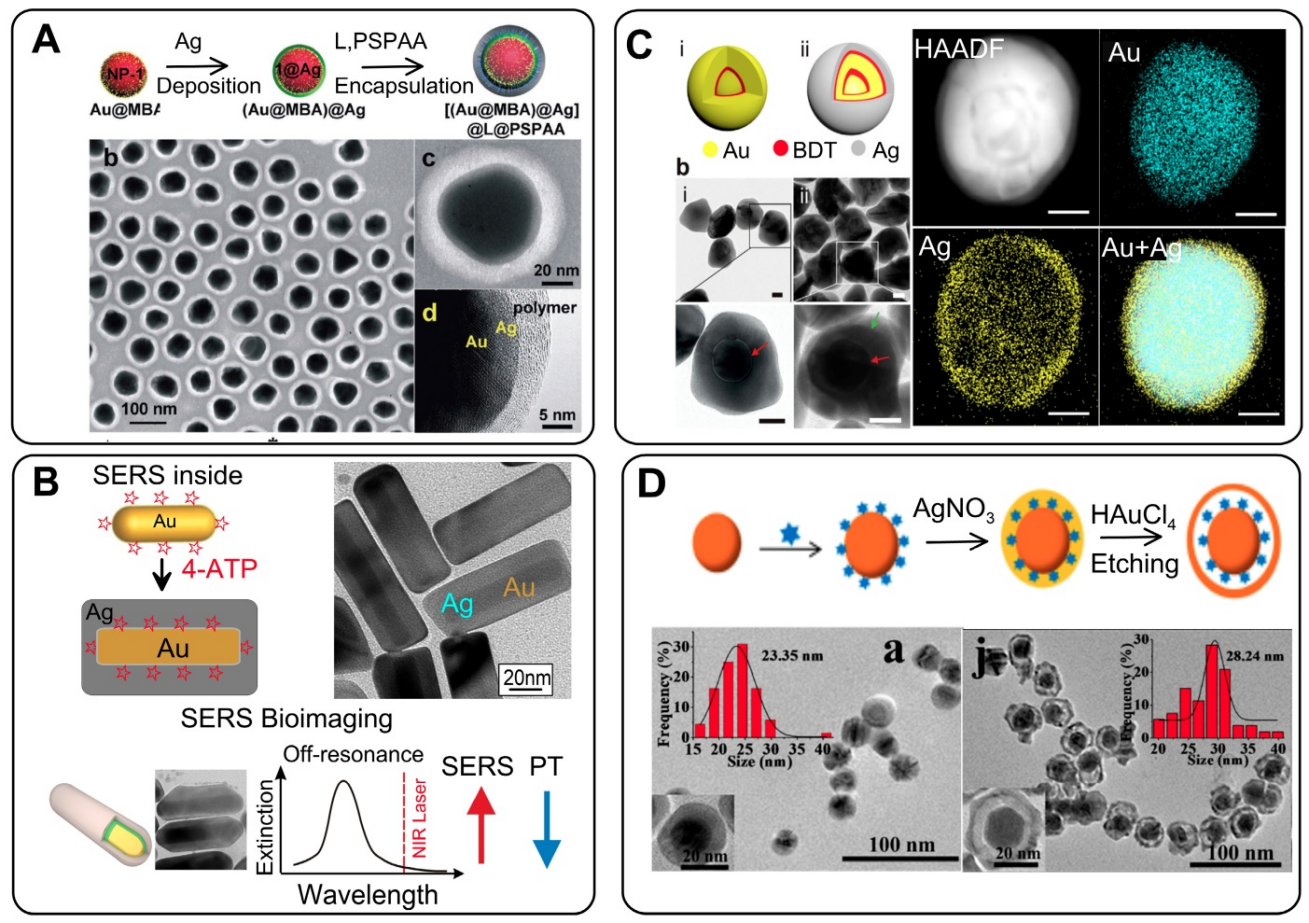
(A) Synthesis of Au@rRM@Ag NPs. Shown also are TEM and HRTEM images of polymer-coated Au@MBA@Ag NPs. Adapted with permission from Ref. 56. (B) Synthesis of AuNR@RM@Ag nanocuboids. HRTEM image of nanocuboids with embedded ATP molecules. Schematic representation of the rational design of SERS and PTT for Ag coated AuNRs. Adapted with permission from Refs. 35, 59. (C) Schematic diagrams and TEM images of single-shell GERTs (Au@BDT@Au) and (ii) bimetallic double-shell GERTs (Au@BDT@Au@BDT@Ag). Shown also is the EDS element mapping of a double-shell GERT for Au, Ag, and the overlaid image. Adapted with permission from Ref. 60. (D) Synthesis of Au@gap@Ag-Au NPs. Representative TEM images of Au@RM@Ag and Au@gap@Ag-Au NPs. Adapted with permission from Ref. 63.

It is well known that in the presence of reporters *in situ* deposition of Ag can enhance SERS signals 54. On the basis of these observations, Xu *et al*. 55 synthesized, for the first time, Au@Ag core@shell NPs by reducing AgNO_3_ around AuNPs functionalized with mercatobenzene acid (MBA). As a result, Raman-active molecules were embedded between the surface of a 16-nm Au core and a 3-nm-thick Ag shell. With increasing shell thickness, the SERS signals from MBA were enhanced by several orders of magnitude. Later, such a synthetic strategy (Figure 5A) was used to prepare different Au@RM@Ag NPs. Different types of RMs such as MBA 56, 4-mercaptopyridine 57, 4-NBT 58, and toluidine-blue-labeled polyacrylic acid 45 have been used together with ascorbic acid and hydroxylamine hydrochloride as Ag reductants.

The covering of Raman-labeled AuNPs with Ag shells has several special features, as compared to the use of Au shells. First, the thickness of the Ag shell can be very accurately controlled by the amount of added Ag precursor. Moreover, it is possible to make 1-nm and even thinner shells. Second, the resulting particles have a relatively smooth surface. Third, in most cases, there is often no gap between the Au core and the Ag shell, regardless of the type of reporters, spacers, and reductants. In any case, such a distinct gap cannot be identified even by HRTEM. This clearly indicates that Au and Ag shells are formed by different mechanisms. As noted above for Au shells, the concept of the formation of bridges on the surface of a particle, with their subsequent closure, is fundamental. On the other hand, for Ag shell formation, the isotropic “overgrowth” of the shell over the entire surface of the particle is obvious. It should be noted that the Au core/Ag shell NPs with internal RMs also fall into the category of GERTs in this review, even if they do not literally exhibit an 'internal gap' owing to their fabrication method. A discussion of such tags is important for two reasons. First, the initial steps of tag synthesis (fabrication of the core and functionalization with RMs) are equal to both Au@RM@Au and Au@RM@Ag tags. Second, the Au core/Ag shell NPs with internal RMs can serve as a template for the formation of gap-based tags by the galvanic replacement reaction.

From a SERS response perspective, there is an optimal thickness of the Ag shell, providing the highest SERS signal. It follows from several experimental observations that the formation of Ag shells on Au spheres rapidly increases SERS at the initial stage, when the Ag shell thickness increases up to 5-6 nm. Then the SERS response gradually decreases 55, 57, 58. Thus, the thickness should be optimal for Au@RM@Ag complexes. For an optimal Ag coating, the maximal SERS response can be increased by 20-50 times, depending on the core size 55, 57. Thus, the absolute value of the SERS signal for Au@Ag is comparable to that of Au@RM@Au nanogapped particles.

Additional advantages can be obtained with nonspherical particles as cores for Au@Ag complexes with incorporated reporters. We investigated the SERS response from ATP molecules adsorbed on the surface of and embedded inside Au@Ag NRs (Figure 5B). For particles with identical sizes and structures, the ATP molecules in the interior showed a strong and uniform SERS intensity, at least one order of magnitude higher than that from ATP adsorbed on the NP surface 35. Because the HRTEM images of Au@ATP@Ag GERTs do no reveal any distinct gaps between the metal layers, the identification of molecule position inside the particle is challenging. To confirm the successful embedding of the ATP molecules, we used the protective properties of the Ag shell and showed that ATP was not oxidized after incubation with hydrogen peroxide 7. Importantly, the ATP molecules inside the Au@Ag NRs showed the highest SERS response under off-resonance laser irradiation, when the laser wavelength was quite far from the plasmon resonance of the NPs 59. This property, together with the tunability of the resonances of Au@Ag NRs by Ag shell thickness, makes possible the rational design of particles for the highest photothermal efficiency or the highest SERS response, without photodamage to the particles and the surrounding objects (Figure 5B).

By analogy with Au@Au multishell NMs, it is possible to use Au@RM@Au GERTs as cores to form double-shell GERT particles (Figure 5C). A study of the dependence of the SERS response of double-shell bimetallic GERTs on the Ag shell thickness showed that NPs with 2-nm-thick shells had the best SERS performance, as well as good photostability 60.

Finally, gap-free Au@RM@Ag NPs can serve as a template for the synthesis of Au@RM@Au-Ag GERTs. The basic idea behind this approach is the galvanic replacement reaction between Ag shell and Au ions 61. Indeed, when Au@Ag NPs serve as the template for a galvanic replacement reaction between Ag and HAuCl_4_, partly hollow Au-Ag alloyed nanostructures are formed (Figure 5D). In this case, the RMs occur in the gap between the Au core and the Au-Ag alloy shell. In general, the NPs with the interior nanogap showed an enhanced SERS signal, as compared to equal-sized NPs without nanogaps 62. For example, Zhao *et al*. 63 reported on a strong SERS response from such GERTs, with the intensity being at least 4 times higher than that for gap-free particles. Clearly, the SERS enhancement of GERTs should depend on the precise structure of the gap and the shell composition. Therefore, further studies are needed to elucidate this point.

## 3. Optical properties of GERTs

### 3.1 Far-field properties

As for any small particles, the far-field properties of GERTs can be described in terms of angular-dependent quantities such as the differential scattering cross-section and the integral absorption, scattering and extinction cross-sections 64 *C_abs_*, *C_sea_*, *C_ext_* = *C_abs_* + *C_sea_* − the total absorbed, scattered, and attenuated EM power [W] normalized to the incident light intensity *I*_0_ [W/cm^2^]. For an arbitrary multilayered structure, the integral cross-section spectra can be accurately calculated by equations that are formally identical to the Mie series for usual homogeneous spherical particles. The only difference is that now the Mie coefficients depend on the GERT structure. At present, there are several efficient numerical algorithms and computer codes to calculate the Mie coefficient for multilayered spherically symmetrical structures 13.

For GERTs with a complex geometry, the integral cross-sections can be calculated with state-of-the-art numerical codes available freely or commercially. In particular, the finite difference time domain method (FDTD, Lumerical Solution), the finite element method (FEM, COMSOL), and the discrete dipole approximation (DDA) are suitable for such purposes (for other numerical techniques, see, e.g., Refs. 65, 66 and references therein).

**Figure 6:**
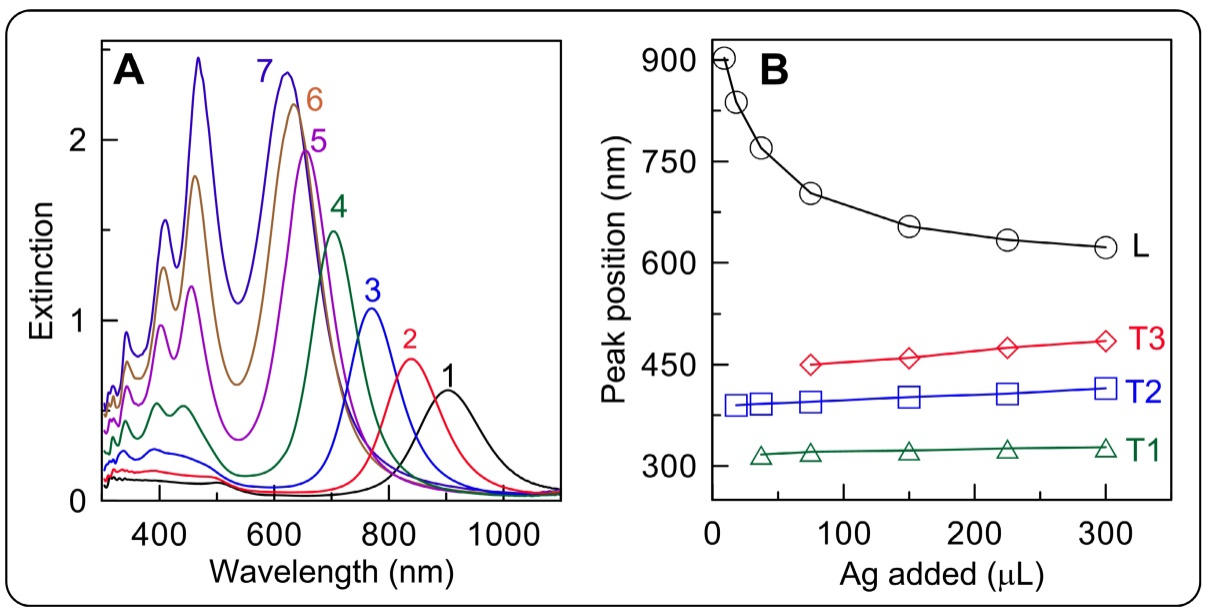
(A) Extinction spectra of the Au@ATP@Ag GERTs with outer Ag shell thicknesses of 0.6 (1), 1.8 (2), 7 (3), 12.5 (4), 15,5 (5), 17 (6), and 19.1 (7) nm. The length and thickness of the initial AuNRs are 72 and 12 nm, respectively. Dependence of the major extinction peak positions for the longitudinal (L) and transversal (T1, T2, T3) modes of Au@Ag cuboids on the added volume of 0.1M AgNO_3_. Adapted with permission from Ref. 35.

For illustrative purposes, we provide the experimental extinction spectra of cuboid GERTs with ATP molecules localized between AuNR surface and silver shell of a variable thickness (0.6-19.1 nm) 35 (Figure 6). Further information about the rational design and far-field properties of anisotropic GERTs can be found in Refs. 35, 59, 67.

### 3.2 Optimal design of spherical Au@Au GERTs

**Figure 7:**
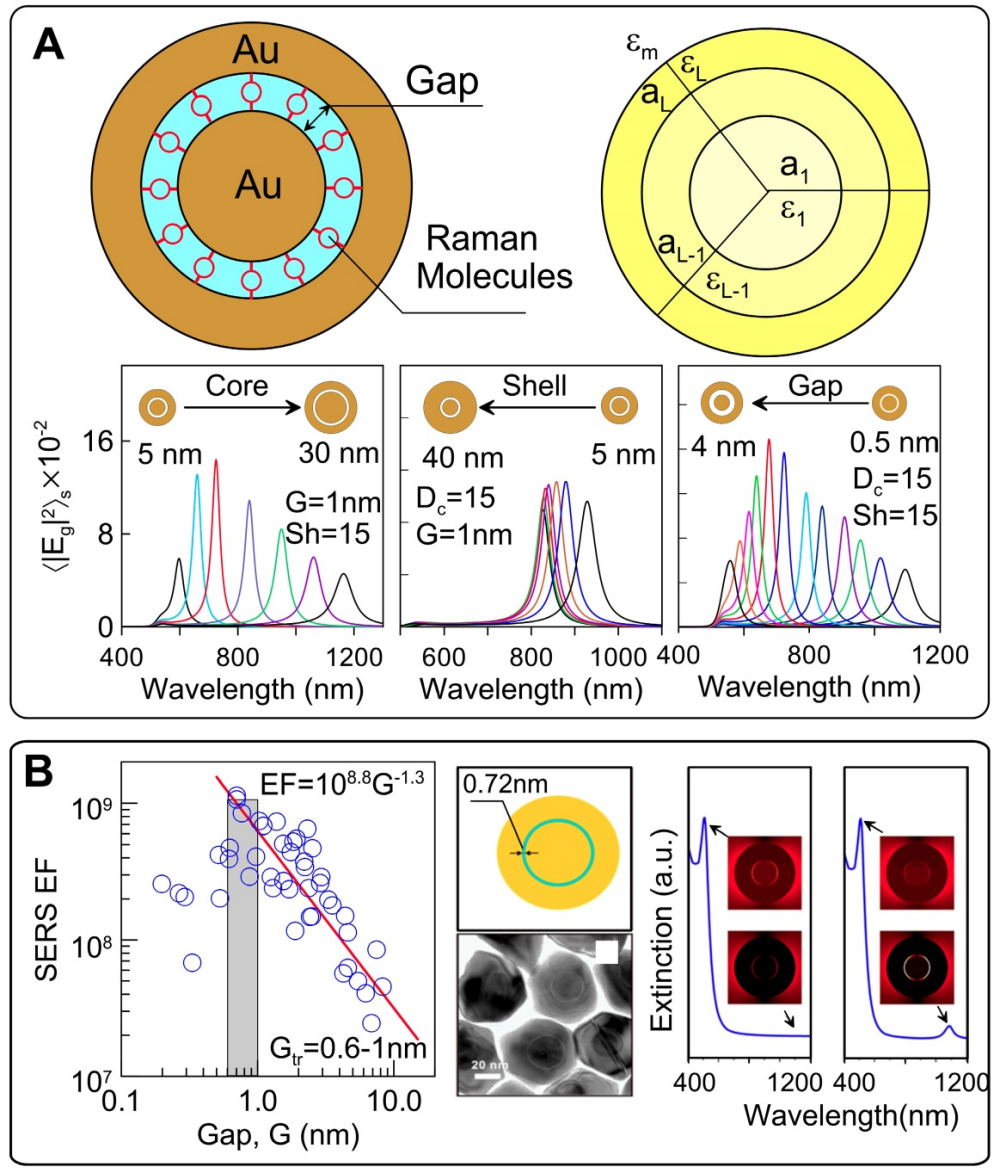
(A) Schematic model of a multilayered Au GERT with embedded RMs. The bottom row shows the spectra of the enhancement factor E^2^, calculated for core diameters of 5-30 nm at a constant gap G = 1 nm and shell thickness S = 15 nm (left), for shell thicknesses of 5-40 nm at a constant gap *G* = 1 nm and a core diameter of 15 nm (center), for different gap thickness *G* = 0.5-8 nm at a constant core diameter of 15 nm and a shell thickness of 15 nm (right). Adapted with permission from Ref. 13. (B) Quantum tunneling effect revealed by measurements of the experimental maximum SERS EFs for 45 Au nanodimers (left). Quantum tunneling effect on the extinction spectra of Au nanomatryoshkas with 0.7-nm interlayers (right). Note the different NIR parts of the classical and quantum-corrected spectra (indicated by arrows). Adapted with permission from Ref. 36.

The SERS signal from GERTs is largely affected by the design of the plasmonic nanostructures. On the basis of numerical and analytical simulations of the EM field inside Au(core)/Gap/Au(shell), we optimized the geometrical parameters of GERTs to obtain the maximal SERS-related parameters for the most popular laser wavelengths of commercial SERS devices 13. To this end, we provide efficient analytical solutions for averaged local field intensities by using exact Mie and approximate dipolar multilayered NM models (Figure 7A). In particular, we provide extensive calculations to explore the spectral dependence of the surface average local field intensities as a function of the core/gap/shell dimensions for typical experimental parameters of Au GERTs.

The peak position wavelength linearly depends on the core size and varies from 600 to 1200 nm. For a gap size of 1 nm and a shell thickness of 15 nm, the maximal EM enhancement was observed for core diameters of 10-20 nm (Figure 7B, left). In contrast, variations in shell thickness give only moderate variations in EM field enhancement and a low spectral dependence 13. We found that for spherical Au@Au GERTs, there exists an optimal gap that ensures the maximal surface average intensity in the gap. In particular, a maximal intensity of about 1700 is observed for a 2-nm gap at an excitation wavelength of about 675 nm. With increasing gap thickness, the enhancement peak moves to the NIR region, as exemplified by the spectra in Figure 7B (right). The most sensitive spectral tuning is observed for gaps in the range 0.5-2.5 nm. For thicker gaps, the spectral tuning is less effective.

For the most common 633 and 785-nm laser excitation wavelengths, the surface averaged intensity in the gap can be about 1000 for the optimal GERT structures. The optimal structure of Au NM for 532 nm seems unrealistic because of the small core size and shell thickness. Importantly, the averaged intensity in the gap is about two orders of magnitude higher than the averaged nearfield intensity around the GERTs. This demonstrates the effective plasmonic concentration of the local field in the gap.

Understanding of the enhancement mechanism is important for the precise prediction and design of functional GERTs for different uses. In the classical electromagnetic model, the plasmonic coupling increases with decreasing gap size in the range of nanometers. This is simply due to the stronger local plasmonic fields generated in the gap. However, for subnanometer size gaps, quantum effects such as electron tunneling *via* the gap between the core and the shell may occur, resulting in a dramatic decrease in the internal field and, therefore, in the SERS response 68. To understand the possible influence of the tunneling across the gap, Zhu *et al*. 69 measured the SERS enhancement for pairs of Au nanodisks with controllable angstrom-scale separation. Those experiments indicated that when the tunneling started playing an important role, the threshold gap value was between 0.6 and 1 nm (Figure 7B). To account for this effect, a quantum-corrected model (QCM) was developed for improved calculations of the electric fields in the subnanometer gaps, where the theoretical results from classical theory are inconsistent with experimental observations (Figure 7B) 36. In this model, electron tunneling across the gap is simulated by replacing the junction with an effective medium characterized by the tunneling conductance 70. Using QCM, we explained some mismatches of the far-field spectra and near-field enhancement between experiments and classical theory. First, the low-energy gap mode in the far-field extinction response predicted by the classical EM model is strongly quenched and unobservable either in experiment or in QCM simulations (Figure 7B, right). Furthermore, the gap size for the maximal experimental near-field enhancement (1.35 nm for 785-nm excitation and 1.8 nm for 633-nm excitation) in GERTs is different from the classical theory (2 nm for 785-nm excitation and 6 nm for 633-nm excitation), whereas the QCM predictions fit well with the experiments. This means that the maximum Raman enhancement is possibly limited by the electron transport between core and shell, which leads to an optimal gap size (i.e., 1.35 nm for 785-nm excitation and 1.8 nm for 633-nm excitation) 71. The results offer a strategy to synthesize GERTs with tunable interior gaps and provide guidelines for their design.

For the understanding of the enhancement mechanism, these studies suggest that the electron transport (i.e., chemical enhancement) certainly plays a role, which is confirmed by the spectral changes in the Raman molecules embedded in GERTs, as compared to Raman spectra in solutions 70. We also believe the EM enhancement should give a large contribution, because only the chemical enhancement may not be sufficient for such high EFs of GERTs. More investigations are needed to understand to what extent the electromagnetic or chemical enhancement effects contribute within the interior nanogaps. We believe that further optical characterization, for example, dark-field scattering spectrum, SERS measurements, and optical modeling on individual GERTs, may offer additional insight into plasmonic electron transport. Previous investigations have demonstrated that electron transport occurs for these 3D intra-particle nanogaps ranging in size from 0.7 to 2 nm. For practical applications, one would aim to achieve a nonquenching system, for example, by further improving Raman enhancement by increasing the gap size beyond 2 nm to reduce electron transport between core and shell.

### 3.3 Influence of gap structure and nanoparticle shape

**Figure 8:**
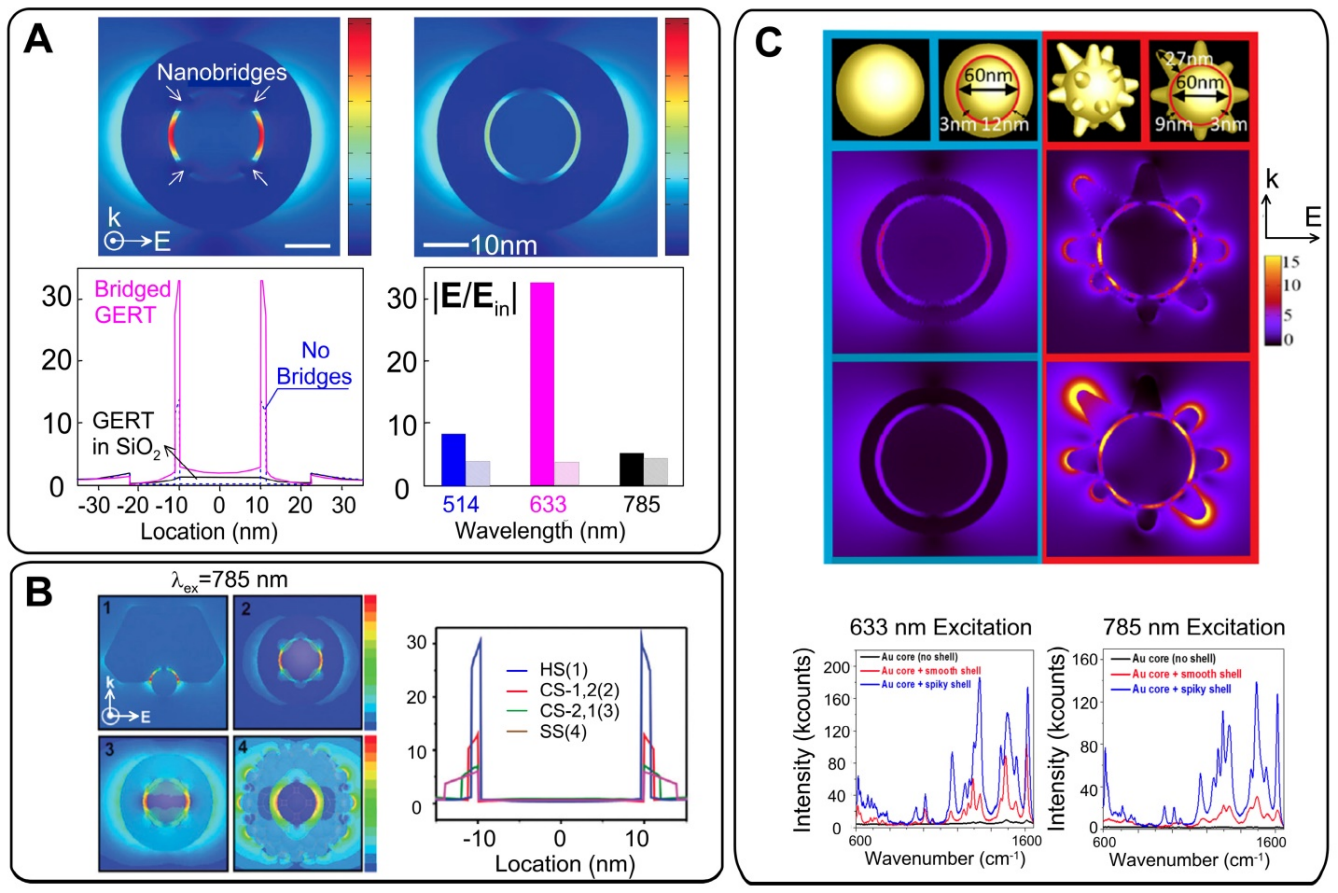
(A) Calculated near-field EM field distribution of bridged and non-bridged GERTs. Comparison of the EM field distribution profiles along the center-horizontal line at an incident wavelength of 633 nm between nanobridged and nonbridged GERTs. The right part shows the wavelength dependence of the field enhancement inside the nanobridged and nonbridged GERTs. Adapted from Ref. 5. (B) EM simulations and the EM field distributions along the center-horizontal line at an incident wavelength of 785 nm for the half-shell NP [HS (1), blue], closed-shell NP with a 1.2 nm intra-nanogap [CS-1.2 (2), red], closed-shell NP with a 2.1 nm intra-nanogap [CS-2.1 (3), green], and star-shaped NP [SS (4), magenta] with an irregular nanogap. Adapted with permission from Ref. 33. (C) Simulated EM field distribution for GERTs with a smooth and spiky surface at an excitation wavelength of 633 nm (top) and 785 nm (bottom). Experimental SERS spectra for the core containing a polymer-conjugated dye (black), a smooth core-shell structure (red), and a spiky core-shell structure (blue), at both 633 and 785-nm excitation wavelengths. Adapted with permission from Ref. 21.

The model discussed above is related to ideal NPs with a spherical core, an ideal uniform gap, and a perfectly smooth spherical shell. In actual practice, the field distribution inside the gap is affected by gap structure and by the morphology of the shell. As discussed above, the shell of the GERTs touches the core surface in some parts and the nanobridged gap is typically observed in TEM images instead of an entirely hollow gap. In a pioneering study, Lim *et al.* 5 showed an enhancement of the local fields in the nanobridged gaps. Theoretical simulation of the field distribution (Figure 8A) shows that the electromagnetic enhancement is highly localized in the interior region of the bridged gap, and the magnitude of the maximal enhanced electric field is ~33 times higher than that of the incident light. However, only a 3.2-fold enhancement was obtained from the hollow gap structure with the same dimension. For the nanobridged gap structure, 633-nm laser irradiation produced the highest signal intensity. Later, the same group showed that the largest increase in the EM field in the nanobridged interior gap was observed at 60° and 90° arc angles of nanobridges for a 633-nm incident wavelength and at 15° and 30° arc angles of nanobridges for a 514-nm incident wavelength 29. For larger nanobridges, the EM field intensity declines quickly. Thus, the nanobridged structure controls the maximal SERS response from GERTs with nanobridged gaps. We also confirmed that the morphology of the core may control the formation of bridged/hollow nanogap inside GERTs and their SERS response 20.

To understand the influence of the shell structure on the SERS response, Lee *et al.* 33 synthesized three types of GERTs (half-shell GERTs with sub-1.0 nm nanogaps, closed-shell GERTs with a wide nanogap (2.1 nm), and star-shaped GERTs with an irregular nanogap (1.5-4.0 nm)). The SERS responses were simulated and measured for these nanostructures under 785-nm laser excitation. The half-shell nanostructures with open sub-1.0-nm intrananogaps showed much stronger SERS responses than did the closed-shall and star-shaped ones (Figure 8B). In addition, the GERTs with open shells demonstrated wavelength-insensitive SERS responses.

The role of the spiky outer surface in Au@Au GERT performance was studied by Jana *et al.* 21. EM simulations and experimental SERS spectra were measured from RMs embedded inside and absorbed to the outer surface. These data were also compared to those obtained for equal-sized smooth GERTs. The SERS enhancement in the region between core and shell was significantly higher for the spiky-shell GERTs. It was found from EM simulations (Figure 8C) that the difference between the smooth and spiky GERTs is larger for 785-nm excitation, as compared to that for 633 nm. Specifically, the simulations predicted an order of magnitude increase in the EM field for the spiky particles at 785-nm excitation and only a 4-fold increase at 633-nm excitation. The findings were confirmed by measurement of the SERS signal from Rose Bengal dye embedded inside two types of nanostructures and adsorbed on the core. When the dye molecules were placed on the outer surface of the spiky GERTs, the SERS enhancement was similar to that observed with Au nanostars of comparable diameter.

### 3.4 SERS enhancement factors, uniformity, and photostability

A typical figure of merit of the SERS response is the fundamental enhancement factor (EF), which is calculated as the ratio of SERS intensity to normal Raman intensity normalized to the number of excited molecules 72. It is now believed that an enhancement factor of the order 10^8^ may be sufficient for single SERS tag detection with common Raman microscopy 73.

Table 1 summarizes the data on the experimentally measured EFs for various Raman reporters embedded in different GERT types.

For GERTs made with DNA-based spacers 5, 27, 31, 32, 48, typical EF values are in the range 10^8^-10^9^. Moreover, the highest EF values were observed under 633-nm laser excitation, whereas under 514-nm and 785-nm excitations, the SERS response was relatively lower. It should be noted that all the above measurements with DNA-based GERTs were performed only by Jwa-Min Nam's group by using a specific technique: AFM-correlated single-particle nano-Raman mapping.

For spherical Au@Ag NPs with embedded Raman-active molecules, the measured EFs lies within a narrow range of (2-5)×10^5^ 45, 60, 74, regardless of the reporter type (thiolated aromatic molecules, dyes, etc). The EF can be increased to 5×10^6^-5×10^7^ by choosing nonspherical AuNR 35 or AuNSt 75 cores instead of spherical ones.

Of note is the work by Gandra *et al.* 18, who reported an outstanding EF value of 1.7×10^11^ for a single Au@BDT@Au GERT. Later, this value was not confirmed by EF calculations for analogous particles 20, 34, 46. The difference between “typical” and “ultra-high” EFs can be attributed to different evaluating the number of exited Raman-active molecules used in EF calculations. In general, the calculated EFs for the molecules embedded in GERTs are one or two orders of magnitude higher than the EFs for the same molecules adsorbed on the NP surface 21, 35. More importantly, GERTs demonstrate higher SERS signals than do other promising SERS tags such as NRs, nanoshells, and nanostars 18, 46.

**Figure 9:**
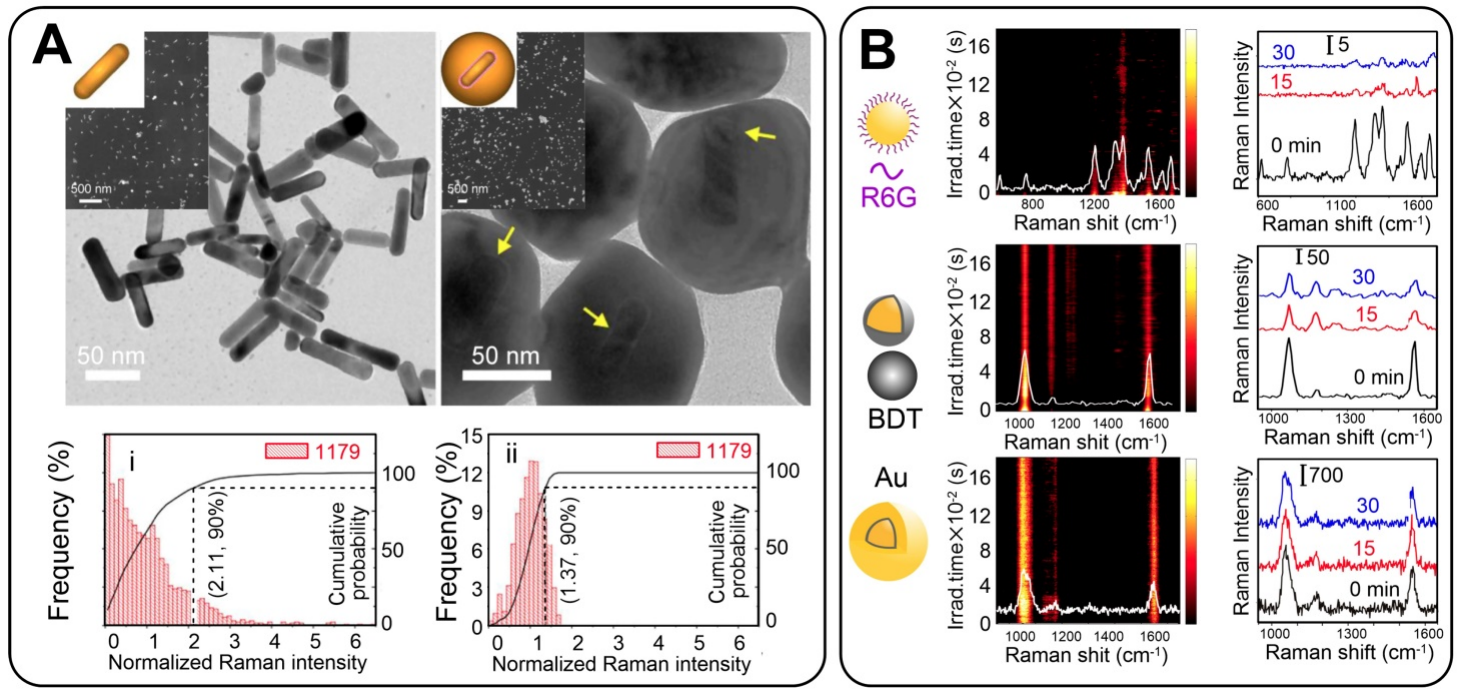
(A) TEM and SEM images of AuNRs and AuNR-based GERTs. Normalized Raman intensity distribution of the Raman band at 1179 cm^-1^ for AuNRs and AuNR-based GERTs. Adapted with permission from Ref. 47. (B) Schematic diagrams, photostability measurements of time-resolved SERS spectra of solid NPs on a silicon wafer during continuous irradiation for 30 min, and three representative SERS spectra at selected irradiation times for AuNS-R6G, AuNS-BDT, and Au@BDT@Au GERTs. Adapted with permission from Ref. 6.

Ideal GERTs should not only provide a high SERS signal but also produce a stable SERS response regardless of particle aggregation and a highly uniform spectral pattern to ensure a linear correlation between the probe concentration and the SERS intensity. Indeed, aggregated plasmonic NPs give a quite high SERS response with an EF of up to 10^10^ because of the large number of hot spots in the contact points of the aggregated particles. However, the random distribution of hot spots resulted in a nonlinear dependence of the SERS signal on tag quantity. This makes the application of NP aggregates challenging in both sensing and imaging. Owing to the core-shell structure of GERTs, the Raman reporters are located inside the particles and are protected by the outer metal shell. We 47 examined the SERS response from individual and aggregated GERTs (based on AuNR core) and also compared the intensity and uniformity of the signals from individual GERTs and aggregated nanorods. AuNR-based GERTs not only generate a stronger SERS signal but also isolate SERS hot spots with Au shells to avoid the influence of the particle aggregation. As a result, such aggregated GERTs showed better SERS uniformity and stronger SERS intensity than did the aggregated normal AuNRs (Figure 9A).

Recent single-particle nano-Raman mapping analysis of DNA-based GERTs revealed that more than 90% of GERTs had EFs greater than 1.0 × 10^8^, which is sufficient for single-molecule detection, and the values were narrowly distributed between 1.0 × 10^8^ and 5.0 × 10^9^, 5, which is mainly due to the uniform interior hollow nanogap structure. We also very recently reported that more than 80% of 34 single-particle measurements produced Raman signals from P-GERTs between 350 and 450 counts (633 nm, ×100 objective, NA = 0.9, 110 μW power, 10 s acquisition time), demonstrating good uniformity at the single-NP level 39. This can be explained by the fact that although the petal-like structures of P-GERTs are random and poorly controlled, each P-GERT has a large number of petal-like structures on its surface, which results in a uniform average Raman signal.

In imaging applications, it typically takes minutes to hours to acquire a Raman image over a large area with SERS NPs. During SERS measurements, photobleaching can occur because of photoinduced heating and possible photochemical reactions. Thus, the photostability of GERTs upon continuous irradiation or in various media is crucial for biomedical imaging and quantitative analysis. In general, a large laser power density and near-field enhancement inevitably lead to undesired amplified photobleaching effects in SERS tags. But GERTs are typically off-resonantly excited by an NIR laser, which allows one to minimize the pronounced photothermal damage to SERS tags and to bring about ultrahigh photostability 6. Furthermore, by hiding Raman reporters in nanogaps and isolation them from the environment (including oxygen, moisture, biological buffer, *etc.*) by an outer metal shell, GERTs avoid the signal fluctuations related to desorption of Raman molecules and to photoinduced chemical reactions. As a result, GERTs demonstrate highly stable spectral patterns with a linear correlation between the probe concentration and the SERS intensity. This is especially important for some susceptible Raman reporters, for example, MBA, which undergo changes in their dissociation state or structure in an ionic or a biological environment, even if obtained with seed-mediated protocols 76. This may lead to a spectral variation for conventional SERS tags in a biological system, which, however, could be avoided in the case of GERTs.

Recently, we compared the photostability of GERTs and common SERS tags decorated on the NP surface 6. The experiment was performed under challenging measurement conditions, under which solid NPs were dried on a silicon wafer and were continuously irradiated with a laser for 30 min at a power density of 4.7 × 10^5^ W/cm^2^. Negligible Raman bleaching or fluctuations were observed with GERT tags whereas common SERS tags had their intensity decreased by 60-90% after irradiation (Figure 9B). The ultra-photostability of GERTs permits them to be used in the high-speed bioimaging of cancer cells and tissues and in other applications with a need for high-power laser irradiation.

### 3.5 Advantages and disadvantages of GERTs

The above analysis of the recent publications on GERTs reveals the main advantages and disadvantages in their biomedical and theranostic applications, which we summarize in this section. The most important advantage is the one or two orders of magnitude higher SERS response from GERTs, as compared with the response from other popular tags such as Au nanorods, SiO_2_/Au nanoshells, Au nanostars, Au nanospheres, and Ag cubes 46. The extra high brightness of the GERTs makes them suitable for single-particle detection and imaging with common Raman microscopes 5, 28. The second important point is the uniform distribution of hot spots within a nanometer-sized gap between plasmonic core and shell 18. This leads to relatively low variations in the SERS signal from particle to particle. Furthermore, the SERS response is practically independent of light polarization and nanoparticle orientation 5. The highly uniform spectral pattern ensures the linear correlation between probe concentration and the SERS intensity. The third important advantage is related to the isolation of SERS hot spots by an outer metal shell, thus avoiding the unwanted influence of particle aggregation. As a result, GERTs demonstrate better SERS uniformity and stronger SERS intensity than the usual SERS tags 47. Additionally, the Raman reporters embedded in the gap between core and shell are protected from the external interference 20. Finally, GERTs can give a strong SERS signal even under off-resonance laser irradiation 59. This is crucial to minimization of tag photobleaching, which typically comes from photoheating and photochemical reactions under laser irradiation 6.

For biomedical applications, it is important to obtain GERTs that are uniform in shape, size, and EF. Unfortunately, these are contradictory requirements, because the more regular and uniform is the GERT structure, the smaller are the EFs typically observed for such GERTs. Therefore, it is difficult to make highly efficient GERTs with good uniformity in geometrical and SERS properties. Nevertheless, one can suggest some useful recipes to achieve the goal of uniformity. A crucial step is to obtain plasmonic cores with maximal uniformity in size and shape. For spherical particles, the best monodisperse cores can be obtained with seed-mediated protocols 20, 25, 77, which produce high-quality hexadecyltrimethylammonium chloride (CTAC)-stabilized Au nanospheres with diameters ranging from 10 to 150 nm. Another instructive example is the use of AuNRs as plasmonic cores for anisotropic GERTs. The current wet seed-mediated technologies allow the synthesis of high-quality NRs with minimal impurities and very narrow distributions in length and width 78. This makes possible the fabrication of anisotropic GERTs with more or less uniform outer Au or Ag shells formed after the absorption of Raman reporters on the NR core 35, 59, 67. Similar approaches can be used to make anisotropic nanorattles and nanocages 79, 80.

Despite their outstanding SERS properties, GERTs are not free from some disadvantages, which can limit their application in theranostics, as compared with the usual tags. First, the protocol of GERT synthesis consists of at least three complex steps (Figure 1). Consequently, the reproducibility of synthesis and the accurate design of tags of appropriate size and shape is challenging. From this point of view, the use of common plasmonic particles with surface-adsorbed RM seems much easier and offers flexible possibilities.

Second, because of the complex core/shell structure, the total size of GERTs is usually above 50 nm. This can limit applications for which a small tag size is critical. Third, for indirect SERS analysis, the fabricated tags should be functionalized with biomolecules. This can be a problem, because as-prepared GERTs are usually stabilized with polymers or surfactants during synthesis. Therefore, GERT functionalization protocols can be complex and time-consuming 46.

## 4. Biomedical applications of GERTs

### 4.1 Biofunctionalization of GERTs

**Figure 10:**
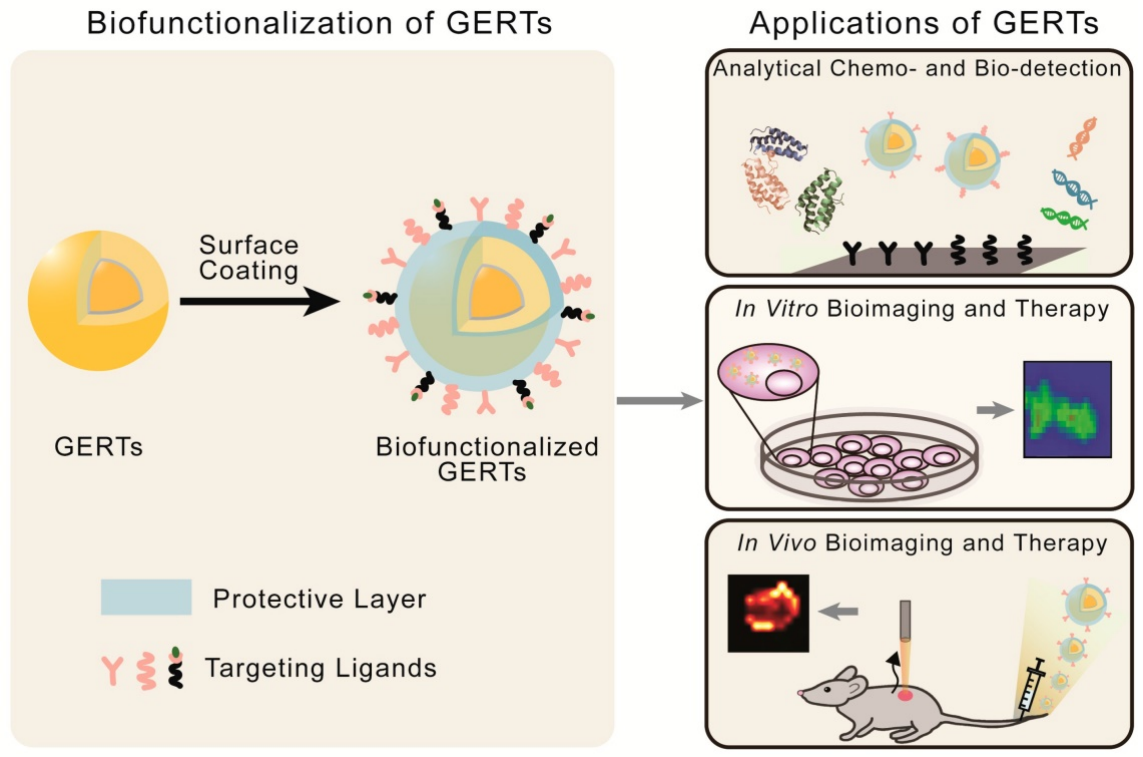
Biofunctionalization and biomedical applications of GERTs.

The SERS-based optical labels applied in biomedicine are called SERS tags (or SERS probes). They consist of metallic NPs, Raman reporters, protective layers, and targeting ligands 7, 8, 81, 82. In such tags, the metallic NPs and Raman reporters are regarded as the basic units providing enhanced and characteristic Raman signals; the protective layers and targeting ligands (surface coatings) are added to render the tags stable, biocompatible, and capable of specific biorecognition. Thus, for application of GERTs in biomedicine, an effective surface coating is needed as a part of bio-functionalization (Figure 10, left panel):

i) **Protective layer.** It (1) enhances the NP stability in solution and NP biocompatibility by replacing the stabilizer or surfactants on the NP surface, (2) provides anchoring sites for the targeting ligands and reduces the nonspecific binding in bioanalysis, and (3) sometimes acts as a matrix to combine other functions, e.g., loading drugs for chemotherapy. The protective layer for GERTs, similarly to that of the common SERS tags, can be chosen from silica (including mesoporous silica) 83, 84, polymers (e.g., polyethylene glycol, polyvinylpyrrolidone, polydiacetylenes) 18, 85, 86, dihydrolipoic acid 51, liposomes 87, 88, bovine serum albumin (BSA) 89-91, and other substances. Among them, silica is one of the most popular materials owing to its easy fabrication and possible removal of some toxic molecules on the NP surface (e.g., CTAC) 8, 11. It is noteworthy that the layer also serves to protect the RMs from directly contacting the outside environments for a common SERS tag whose Raman reporters are located on the metal surface. In this case, the competitive adsorption between protective layers and Raman reporters should be taken into consideration, especially when use is made of polymers with thiol-ends as the protective layers. However, for the core-shell GERTs whose Raman reporters are hidden inside, there is no such requirement, which simplifies the surface modification process. It is also possible for some GERTs to just jump over this step with direct attachment of thiolated biomolecules as targeting ligands to the NP surface 62.

ii) **Targeting ligands.** They are conjugated to GERTs after the modification of the protective layers, and aim to enhance the specific recognition and targeting of analytes. The selection of targeting molecules is similar to that for NP-based agents, which includes proteins, nucleic acids, aptamers and small biomolecules, depending on the types of analytes 27, 46, 92, 93. The commonly applied functionalization approaches include EDC/NHS conjugation for proteins, and Au-S bonding for thiolated oligonucleotides 62. The number, affinity, and orientations of the conjugated ligands largely influence the specificity of GERTs.

With the properties of high brightness, stability, specificity, and biocompatibility, these biofunctionalized GERTs facilitate the development of new biomedical applications in sensing, imaging, and therapeutics. In this review, we will focus on three classes of theranostic applications (Figure 10, right panel): analytical biodetection, *in vitro* imaging and therapy, and *in vivo* imaging and therapy.

### 4.2 Analytical chemo- and biodetection

#### 4.2.1 Detection of bioactive molecules

The quantitative determination of bioactive molecules, including proteins, nucleic acids, aptamers and small molecules, are the common goals in biodetection assays. In SERS-tag-based biodetection procedures, a sandwich assay is widely adopted. The analyte is “sandwiched” between two targeting sites; typically, the targeting site (such as antibodies or complementary nucleic acids) are first immobilized onto the substrates; the target biomarkers are then captured by them, and the biofunctionalized GERTs are attached through the recognition of the biomarkers onto the substrate. This gives a distinct signal for quantitative analysis. GERTs are advantageous as optical labels in the biodetection assay owing to their superhigh sensitivity (down to a single-NP level) and specificity, leading to an improved limit of detection (LOD), as compared to that obtained with common SERS tags. More importantly, the embedded Raman reporters can be considered an internal standard for the calibration of Raman signal fluctuations induced by different measurement conditions and local states of the NPs, such as aggregation. This can greatly overcome the reproducibility issue in common quantitative SERS-based biodetection 94.

**Figure 11:**
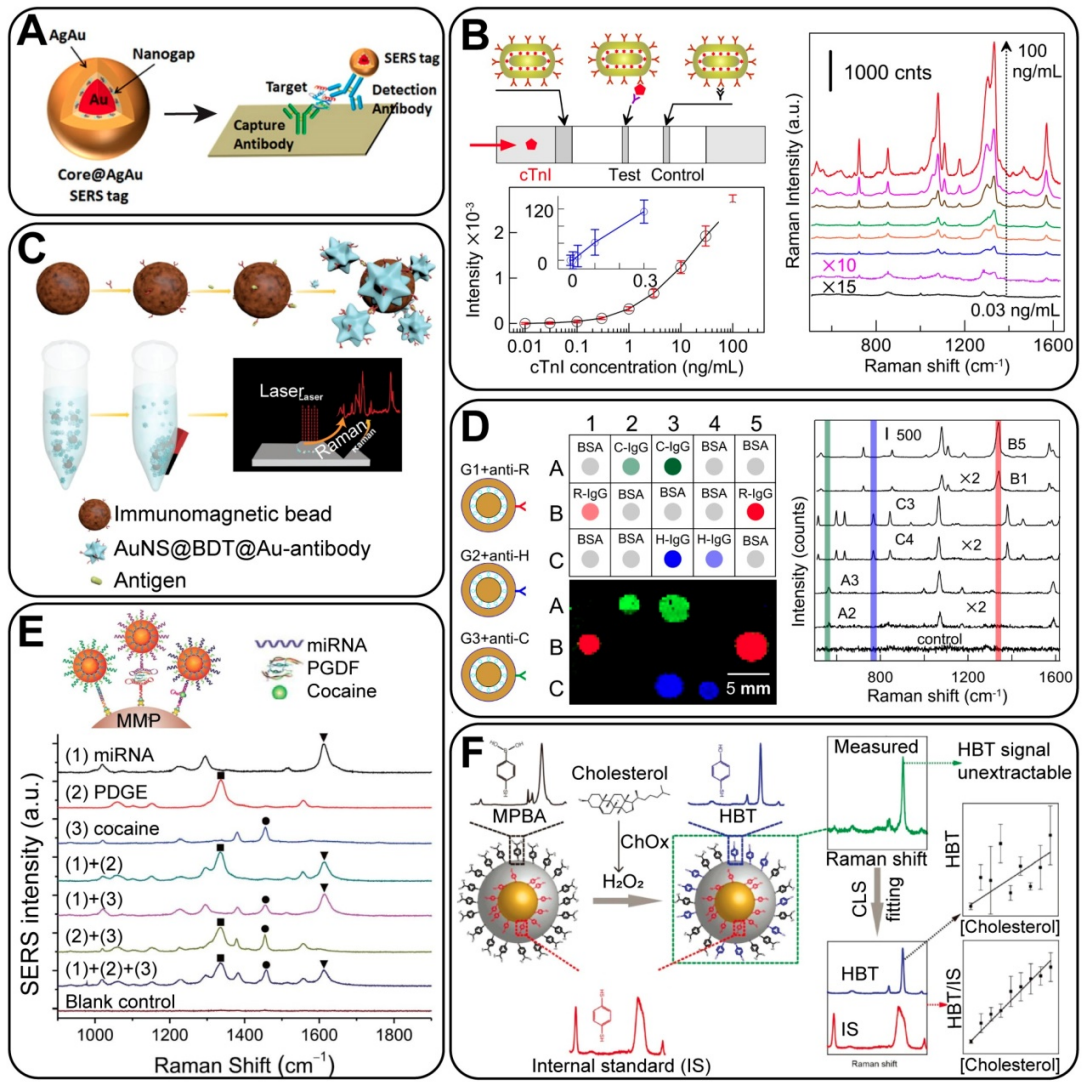
(A) Typical sandwich assay for the detection of an antigen on a 96-well planar substrate by using GERTs. Adapted with permission from Ref. 92. (B) GERT-based LFIA for cTnI, the average SERS spectra in the test zones, and the standard curve of different cTnI concentrations. Adapted with permission from Ref. 46. (C) Schematic illustration of GERT-based immunoassay for biomarker recognition in liquid phase. Adapted with permission from Ref. 51. (D) Multiplex dot immunoassay of GERTs to detect different types of IgG. Adapted with permission from Ref. 98. (E) A multiplex analytical strategy based on GERTs for detecting three types of biomarkers, including microRNA-141, platelet-derived growth factor (PDGF), and cocaine. Adapted with permission from Ref. 27. (F) GERTs with internal standards for the quantitative detection of cholesterol. Adapted with permission from Ref. 94.

SERS-based quantitative detection can be divided into two categories on the basis of the assay platform: detection on a solid substrate and in a liquid phase. For the former, a variety of substrates can be applied, including glass plates 95, quartz substrates 84, paper strips 96, and multi-well plates 92. Random aggregates of NPs on solid platforms are nearly inevitable, thus making problematic the use of common SERS NPs with Raman reporters on their surface for quantitative analysis. This is because the nanogaps from the NP aggregates may produce uncontrollable and irreproducible SERS hot spots. However, GERTs are beneficial in this case because of their hot spots from the interior nanogaps instead of interparticle nanogaps and because of highly uniform and reproducible Raman enhancement. For instance, Li *et al*. 92 fabricated Au core@AuAg shell GERTs to show a smaller deviation and a much higher signal than that from aggregated AuNPs. In their work, streptavidin-capped GERTs were used in a quantitative enzyme-linked immunosorbent assay on a planar platform (Figure 11A). The detection limit of prostate-specific antigen (PSA) or c-reactive protein (CRP) was ~1 or 3 orders of magnitude lower than that obtained in clinical settings, respectively. This work demonstrates that GERTs have great potential to be integrated with SERS detectors for highly sensitive diagnostics. Recently, we have reported the successful application of GERT-based lateral flow immunoassay (LFIA), either using anisotropic rod-like GERTs (Figure 11B) 46 or using bimetallic double-shell structured GERTs 60. The sensitivity of the former assay highly surpassed that of colorimetric LFIA, with an LOD for cardiac troponin I (cTnI) of 0.1 ng/mL, close to the diagnostic criteria for blood serum in the case of heart infarction. The latter has reached an LOD of 0.025 mIU human chorionic gonadotropin (HCG), which is three orders of magnitude better than the commercial strips. Note that the Raman mapping data in the test zone of the strips can be calibrated by the conditional least-squares method, to further eliminate the background interference of the nitrocellulose membrane and improve the LOD 97. Considering the current popularity of commercial LFIA strips, these works have demonstrated the bright future of GERT-based point-of-care tests, with popularized use of portable Raman systems in the future.

For the assay in a liquid phase, microbeads are the common choice as the platforms. Yang *et al*. 51 have developed GERTs (AuNS@tag@shell particles) by embedding the RMs between nanostars and Au shells. The bright SERS tags were modified with antibody and then attached to magnetic beads through the recognition of antigens (Figure 11C). By measuring the Raman intensity of the bead-SERS tag compounds, an LOD for mouse IgG antigens of down to 0.1 pg mL^-1^ was achieved. Nucleic acids can be detected by a similar sandwich-hybridization assay. Kim *et al*. reported Au core-Ag shell GERTs with uniform built-in nanogaps of ~ 2 nm and embedded Raman reporters, which exhibited a narrowly distributed EF of up to 10^9^ 62. DNA-modified magnetic microparticles and GERTs were used to detect the DNA strands of the target hepatitis A virus (HAV). An ultrasensitive detection of 10-100 aM was obtained, which was reported as 10-1000-fold higher sensitivity than that obtained previously 84. These results confirmed that GERTs can be useful in the ultrasensitive, target-selective, and quantitative detection of biomarkers in liquid samples.

In summary, a variety of GERTs and recognition units have been used in the detection of biomarkers. Table 2 lists comparative data on the assay substrates, GERT structures, Raman reporters, targeting ligands, biomarkers, and detection limits.

#### 4.2.2 Multiplex detection of biomarkers

High-throughput detection has garnered much attention owing to the increasing demand for the simultaneous analysis of multiple analytes. Owing to the characteristics of Raman spectroscopy (the fingerprint spectrum with narrow bandwidth), SERS-based analysis plays an important role in the optical encoding for multiplex detection. For GERTs, different RMs with various functional endgroups can be embedded in the nanogaps to fabricate NPs with variant signals, making them ideal multiplex probes 51.

We reported a dot-immunoassay of three types of IgG by taking advantage of the unique distinct spectral peaks of three GERTs (Figure 11D) 98. This proves that GERTs are general and useful platforms for the multiplex detection of analytes through spectral identification. Similarly, Zhao *et al*. developed a direct synthesis of GERTs by anchoring block DNA and nonfluorescent molecules as Raman reporters in the nanogaps 27. Based on these tags, a quantitative multiplex analysis of virus gene DNA was then performed, with an impressive LOD of down to pM. Also, they provided an analytical strategy to simultaneously detect different types of analytes, including nucleic acids, proteins, and small molecules (Figure 11E). Through this work, GERTs have held promise as multianalysis probes for recognizing the major types of bioactive markers.

#### 4.2.3 Quantitative detection using internal standard calibrations

SERS-based approaches in bioanalysis fall into two main categories: indirect detection, in which SERS tags serve as labels, and direct identification of the vibration information of molecules by using SERS substrates. The concept of GERTs was developed towards the sensitive and quantitative *indirect* detection and imaging techniques, which is relatively similar to fluorescence achieved bioanalysis. It can also be noted that the outer surface of GERTs is a suitable platform for the *direct* SERS analysis of adsorbed molecules, with the advantage of using such embedded reporters as internal calibration standards.

SERS, as an ultrasensitive technique for trace analysis, has not yet been established as a routine method for quantitative analysis because of its poor signal reproducibility. To circumvent this drawback, one strategy is to fabricate uniform SERS substrates to avoid the random distribution of EM hot spots. Another approach is to use the signal of the internal standard RMs to eliminate the interference caused by the NP concentration change. As we have discussed before, GERTs have brought great advances as ultra-stable optical labels in quantitative studies owing to their intrinsic core-shell structure, which avoids the desorption of embedded molecules and the disturbance from the solvent environment or possible aggregation. In this case, the molecules inside nanotags serve as an internal reference, while the outer Ag surface is suitable for label-free SERS detection of analytes. For example, Vo-Dinh *et al.* 75 reported an analytical approach with the internal reference containing silver-embedded gold nanostars. The calculated external EF was 4.1×10^4^, while the internal EF was two orders of magnitude higher (4.2×10^6^). The inclusion of 4-MBA as an internal reference significantly improved the reliability and quality of the linear fitting for the quantification of the analyte concentrations. The same results were further demonstrated by using MBA-embedded Au@Ag nanocubes as a platform with the internal reference signal 99. Shen *et al*. 57 successfully developed a quantitative SERS-based analysis using GERTs, in which the embedding molecules were the internal standards, whereas the targeted molecules were designed to be attached to the outer surface of the shells. The main advantage of such an approach is that the Raman signal of the targets can be effectively calibrated by the internal standard to eliminate the signal fluctuation induced through variations in NP aggregation states and the measurement conditions. This proves that GERTs are reliable platform for the quantitative analysis of a broad range of analytes.

There are several advantages of using GERTs as internal calibration standards: 1) the standard molecules and targets have separate spatial distribution; thus, no competitive adsorption or replacement occurs; (2) the plasmonic core-shell NPs serve not only as a standard calibration but also as a SERS substrate to enhance the signal of targets on the surface; (3) there is a high flexibility in selecting Raman probes and the synthetic route for cores and shells. Note that the embedded standard molecules can be selectively picked to avoid peak overlapping with targets. Still, the spectral fitting methods could be applied to extract quantitative information of each component from the Raman spectra even if there is much peak overlapping, making GERTs applicable to a broad range of analytes. Recently, we adopted Au/Ag GERTs with embedded 1,4-BDT as the internal calibration standard to detect hydrogen peroxide (H_2_O_2_) and cholesterol (Figure 11F) 94. 4-Mercaptophenylboronic acid (4-MPBA) was immobilized on the shell surface (Au@BDT@Ag@MPBA) as the probe, which would react with H_2_O_2_ and changes to 4-hydroxybenzenethiol (4-HBT), inducing a change in the SERS signal of the NPs. By calibrating the relative intensity of 4-HBT/1,4-BDT by the CLS fitting method, H_2_O_2_ was determined with an LOD of 10^-6^ M in PBS solution. Furthermore, these NPs can be coupled with cholesterol oxidase (ChOx), to quantify intracellular cholesterol via H_2_O_2_ produced during the oxidation of cholesterol. That work demonstrated for the first time the quantitative SERS mapping of intracellular H_2_O_2_ and cholesterol at the single-cell level, showing the new potential of reliable SERS analysis by using GERTs.

In addition to *in vitro* quantitative analysis, *in vivo* quantitative analysis using GERTs is appealing. The complicated environment *in vivo* requires high stability of NPs and Raman molecules. It is also critical to distinguish signals of SERS tags from those of biological tissues. Therefore, we consider GERTs as suitable candidates in such a situation. By hiding Raman reporters in the nanogap, GERTs protect the molecules from desorption or interference of their environment* in vivo*, leading to highly photostable signals. Unique Raman signals can be obtained by choosing suitable embedded Raman molecules. In addition, GERTs with an internal calibration standard can be employed as a quantitative biosensing platform to detect biomolecules (such as cholesterol) *in vivo* in a similar way to that *in vitro* 94.

### 4.3 *In vitro* bioimaging and therapy

#### 4.3.1 Cell bioimaging

**Figure 12:**
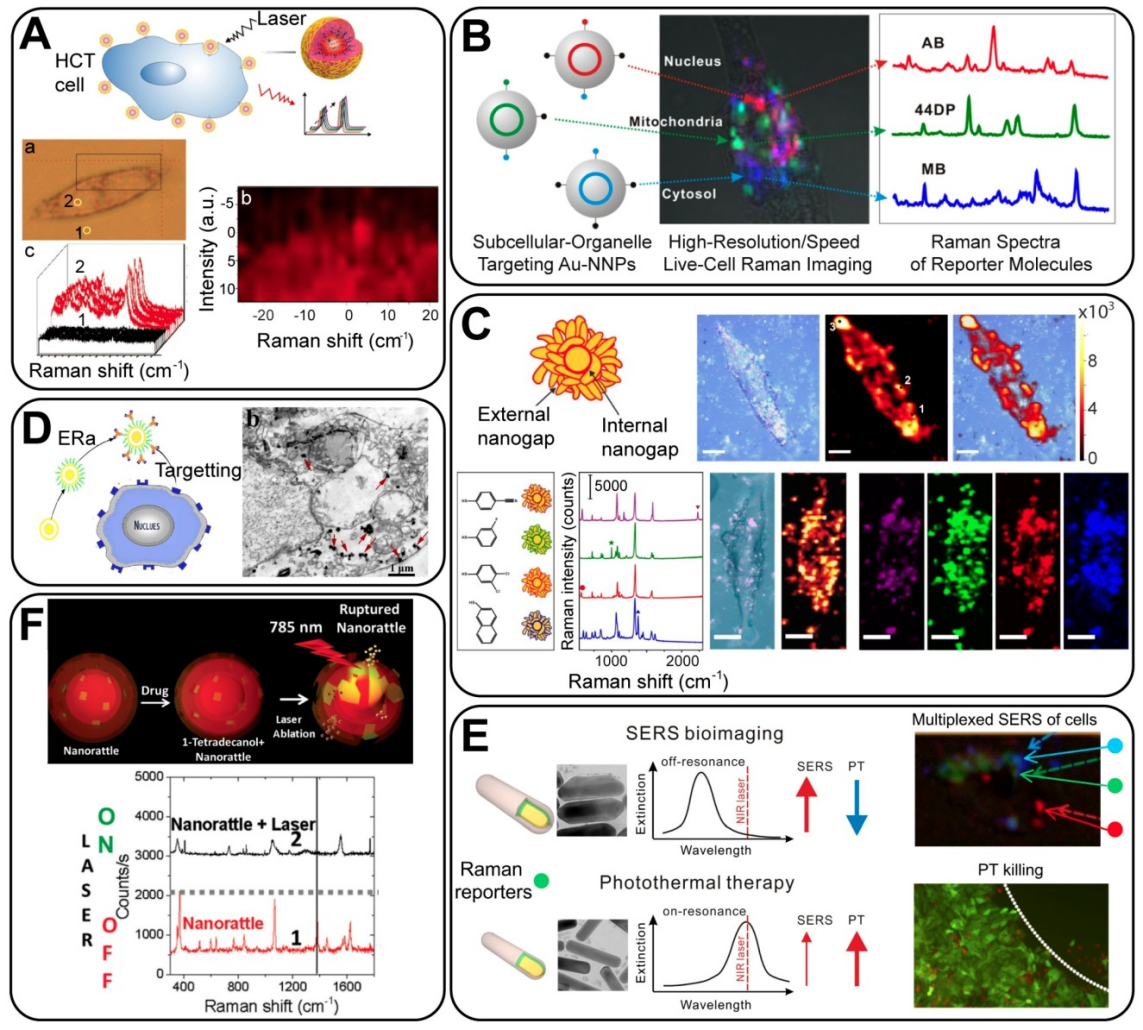
(A) Raman spectra and mappings of GERTs distributed on a single cell surface. Adapted with permission from Ref. 32. (B) GERTs for the high-resolution and high-speed multiplex imaging on a live cell. Adapted with permission from Ref 28. (C) Mesoporous-silica-coated first- and second- generation GERTs for high photostable and high-speed imaging. Adapted with permission from Ref. 39. (D) SERS detection and PTT of cancer cells by using targeted GERTs. Adapted with permission from Ref. 103. (E) The design of GERTs for bioimaging and PTT: off-resonant tags with a thick shell have a high SERS signal and a reduced PT effect, and on-resonant tags with a thin Ag shell show moderate SERS performance and an enhanced PT effect. Adapted with permission from Ref. 59. (F) Raman-guided locoregional therapy using double-shell GERTs. Reprinted with permission from Ref. 79.

As a widely applied biotechnique, *in vitro* cell bioimaging can provide detailed information on the cellular organelles, interactions, and dynamics, and thus, it plays a critical part in fundamental biomedical studies. In recent years, GERTs have been employed for *in vitro* cell bioimaging in a series of studies 8, 18, 28, 32, 33, 62, 92, 100. Apart from being more biocompatible and photostable than fluorophores, the GERTs have high specificity and efficiency of targeting owing to biofunctionalization. As shown in Figure 12A, efficient tumor cell detection using GERTs was reported by Hu *et al*. 32. Also, they showed the possibility of simultaneous duplex determination of biomarkers on the cell surface by using GERTs, which prove a promising candidate for the simultaneous bioimaging and SERS detection of cancer.

Still, the most serious impediment to the general application of SERS-based cell imaging is the limited imaging speed, especially as compared to the widely used fluorescence imaging. The relatively long exposure time on cells (usually in seconds on each pixel) may not only damage the biological samples but also impede the imaging of live cells with a high temporal resolution. To circumvent this problem, many endeavors have been devoted in two directions. One is to improve the Raman systems for high-speed and wide-field imaging, by reducing either the scanning or data collecting time. Gambhir's group 101 reported a small-animal Raman imaging system with a speed of 1.5 min for a 5 × 5 mm^2^ area, showing a 10-fold and a 240-fold improvement in the scan time, as compared with the Streamline mode (Renishaw) and the normal scan mode, respectively. Similarly, Heller's group reported a confocal Raman microscope capable of imaging with a 50-ms integration time per pixel (30 × 30 spectra from 17 μm ×17 μm area) for single-cell imaging 102.

The other strategy is to develop more sensitive SERS tags, among which GERTs hold great promise. By combining both advancements in Raman instruments and SERS tags, Kang *et al.* 28 developed a high-speed SERS imaging system using DNA-mediated GERTs and galvanometer mirror-equipped custom-built confocal Raman microscopy. They successfully achieved single live cell Raman imaging with high speed (30 s, 10 ms/pixel) and high resolution (50 × 50 pixels) under 785-nm laser excitation. Their results also showed the capabilities of GERTs in multiplexing live-cell imaging and in subcellular organelle targeting (Figure 12B). This work may open new possibilities for Raman-based high speed, high throughput, and high content imaging.

We have conducted a series of work on high-speed Raman imaging by using mesoporous-silica-coated GERTs. Two generations of NPs were developed and utilized. The first-generation GERTs were spherical core-shell NPs with embedded thiolated aromatic molecules, which exhibited high storage/pH stability, good photostability, strong Raman signals, and favorable biocompatibility 8. They allowed ultrastable imaging under 30-min laser irradiation at a high power of 10^5^ W/cm^2^, as well as a fast imaging of cells (down to 1 ms/pixel). Very recently, a new version of GERTs (P-GERTs) with petal-like Au shells has been developed. With internal and external nanogaps, these second-generation GERTs show an EF over 10^9^ and a single-NP detection sensitivity, with which the high-resolution cell imaging of 6 s on an area of 50 × 50 pixels under 370 μw laser was achieved (Figure 12C). By further modifying different RMs in the external nanogaps of these GERTs, the high-resolution multiplex cell imaging was performed as well (Figure 12C). These finds enable the utilization of GERTs for high-speed, long-term, real-time and multiplex bioimaging under the continuous laser excitation, showing bright prospects for Raman-based clinical imaging.

#### 4.3.2 Cancer cell therapy

Plasmonic NPs can be useful in the *in vitro* therapy of cancer through their photothermal effect. GERTs are suitable for photothermal therapy (PTT) because of their good dispensability, good photostability, tumor targeting ability, and efficient photothermal conversion. For example, Chen *et al.* 103 demonstrated SERS-guided PTT of cancer cells *in vitro* by using crystalline GERTs (Figure 12D), offering a simple yet powerful design of SERS-based PTT agents. Additionally, as core-shell NPs, GERTs can be designed in two roles: only as bioimaging probes, with minimized photothermal influence on biological samples during the imaging process, or as multifunctional theranostic probes for simultaneous imaging and PTT. This was demonstrated by making plasmonic AuNR@Ag shell GERTs 59 by tuning the Ag shell thickness; the GERTs showed controllable photothermal properties under laser irradiation. They can serve as multifunctional theranostic probes when thinner Ag shells are used (on resonance) or as super-contrast NIR agents for bioimaging with reduced photothermal influence when thick Ag shells are used (off resonance) (Figure 12E). Through the rational design of GERTs with a tunable SERS enhancement and a tailorable PT effect, this work provides a novel strategy for SERS-based cancer diagnostics and therapy.

Moreover, by exploiting the core-shell structure of GERTs, both Raman reporters and chemotherapy drugs can be loaded within the nanogap to achieve a combined effect of chemotherapeutics and Raman-based drug release monitoring. As designed by Gandra et al., double-shell GERTs were modified with two Raman reporters on the Au core and the inner shell, respectively 79. The phase change material (1-tetradecanol) and the chemotherapy drug doxorubicin were loaded between the inner and the outer shell, forming a multifunctional agent (Figure 12F). The significant photothermal effect under laser irradiation would lead to the rupture of the outer shell and thus would release the drug, achieving locoregional PTT chemotherapy, during which the therapy process was monitored by the change in the relative Raman intensity. This work provides a new strategy to design therapeutic agents in both drug delivery and photothermal contrast, opening new possibilities in SERS image-guided therapy.

### 4.4 *In vivo* bioimaging and therapy

#### 4.4.1 *In vivo* bioimaging

**Figure 13:**
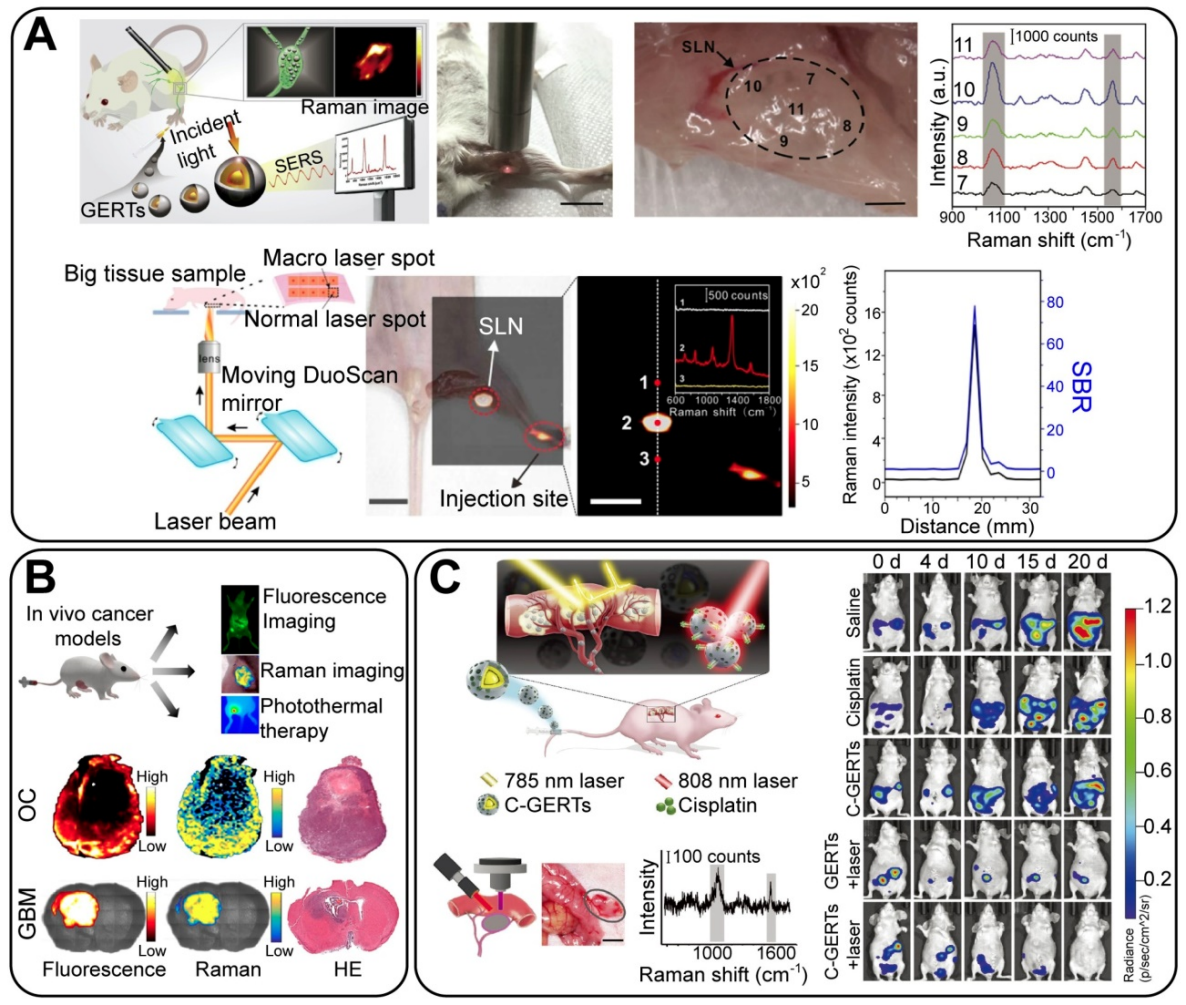
(A) (top) Detection of a sentinel lymph node with a portable Raman spectroscopy scanner by using the first-generation GERTs (S-GERTs). Adapted with permission from Ref. 110. (bottom) High-contrast and wide-area lymph node imaging *in vivo* by using P-GERTs. Adapted with permission from Ref. 39. (B) The SERS-fluorescent NPs for dual-mode cancer imaging and photothermal therapy. Adapted with permission from Ref. 111. (C) SERS-guided detection and chemo-photothermal therapy of abdominal disseminated microtumors in mice by using GERTs loaded with cisplatin. Adapted with permission from Ref. 113.

*In vivo* imaging serves as a bridge between the fundamental studies on cellular dynamics and the clinical use of tumor detection. Following the pioneering work in which SERS tags were used for intraoperative tumor imaging 104, 105, much progress has been made in recent decades, and different SERS tags have been developed such as AuNPs 106, 107, nanostars 108, nanorods 109, and GERTs 6. Bao *et al*. 110 reported the application of untargeted GERTs for the intraoperative imaging of sentinel lymph nodes (SLNs) (Figure 13A, top). These NPs show benefits such as great brightness, good photostability, long retention time in the SLNs (24 h), convenient injection (2 h prior to surgery), and high imaging depth (2 mm); It is also possible to apply a portable Raman spectrometer to locate the SLNs when GERTs are used as the imaging agents (Figure 13A). This proves a promising potential for preclinical applications, given that Raman endoscopes are already in clinical trials 107. Recently, we used P-GERTs with petal-like shells to conduct high-contrast and wide-area *in vivo* imaging on mouse lymph nodes (Figures 13A, bottom). Briefly, the SERS image was obtained in 52 s (20 × 20 pixels) with a laser power of 370 μW and an acquisition time of 0.7 ms per pixel on an area of 3.2 × 2.8 cm^2^. These nanotags also enable the conduct of extremely high-contrast Raman imaging with a signal-to-background ratio of up to ~80, − at least one order of magnitude higher than that of conventional Raman imaging. These results have undoubtedly shown promising future for GERTs as super-bright and stable probes for rapid biosensing and bioimaging applications.

Another strategy to extend the applicable scope of Raman probes is to integrate the fluorescence and Raman imaging techniques, as reported by Pal *et al*. 111. They proposed optimized nanorod-GERTs, using NIR fluorophores with high Raman cross-sections as embedding molecules. These GERTs served as enhanced fluorescent and Raman probes. Cancer tissues in either a subcutaneous ovarian cancer or a glioblastoma (GBM) mouse model were exactly localized by dual-mode imaging (Figure 13B). Pal *et al*.'s work would possibly give rise to attention to the integrated fluorescence-Raman technique in cancer imaging and surgery.

#### 4.4.2 SERS-guided surgery

Despite the major advances in targeted drug and radiation therapies, surgery is still the most preferred and effective treatment for localized tumors 11. Precision cancer surgery guided by intraoperative optical imaging is of broad interest in engineering and medicine. In the past decade, many efforts have been made to optimize the performance of SERS-guided surgery by three strategies: developing superior SERS tags, achieving higher specificity in delivery, and improving the speed by optimizing the Raman system and the imaging methodology. When it comes to a more complete resection of the primary tumor, draining lymph nodes and metastatic sites, superior SERS is required to be of high brightness, good targeting capability, and specificity 11. From this point of view, GERTs can be promising probes. Several groups, including our own, have reported on GERT-guided surgery and cancer therapy. The 1,4-BDT-embedded GERTs developed by us had a detection limit in a liquid of 20 fM, with a laser energy of 10^5^ W/cm^2^ and an integration time of 1.86 s 112. The high brightness makes it possible to conduct real-time intraoperative sensitive detection and treatment of residual prostate tumors 112, as well as to diagnose disseminated ovarian cancers (Figure 13C) 113. These tags can specifically identify and eliminate the tumor margin and microsatellite metastases, making it possible to have a complete tumor resection. Furthermore, loading of the chemotherapy drug cisplatin within the mesoporous silica layer of the GERTs, enables tumor cells to be killed by Raman-guided chemo-photothermal synergistic therapy in a precise way (Figure 13C) 113. Those investigations unveil the attractiveness of GERTs as a robust platform for the intraoperative diagnosis and eradication of microtumors, which would push Raman technologies toward deep theranostic and related biomedical applications.

## 5. Concluding remarks and outlook

We have reviewed the progress made in the fabrication, optical properties studies, and theranostic applications of a novel type of SERS tags - GERTs. As emerging nanotags, GERTs have been synthesized by different synthetic methods, by using common plasmonic materials (Au, Ag) and a variety of Raman reporters such as DNA strands labelled with fluorescent dyes, thiolated aromatic molecules, and Raman-active polymers. We have discussed why the core-shell structure and build-in nanogap benefit plasmonic NPs as a superior SERS tags and how this leads to attracting optical properties and helps promote research on biological detection, imaging, and clinical therapeutics. Some impressive biomedical applications of GERTs have been briefly described.

Despite the great advances made in the fabrication of GERTs by many groups all over the world, the big challenge lies in the chemistry and control of nanogap formation. Previous studies have reported successful gap-size control through the tuning of the thickness of the insulation layer 37, 40 or through the control of the kinetics of the galvanic reaction between Au shell and Ag middle layer 114. Still, more fundamental studies are lacking, given that synthetic manipulations with large number of particles on the nanoscale are of critical importance for successful biomedical applications in the next step. Investigations into the enhancement mechanism of GERTs should make us understand how the plasmons on core and shell interact with each other and to what extent that influences the EM or chemical enhancement within the interior nanogaps. The mechanism, however, varies from particles to particles owing to the differences in the gap size, material, type of RMs and synthesis route. Precise control of nanogap size opens new possibilities for the design and use of GERTs in specific applications.

The sensitivity of GERTs is very important in analytical, bioimaging, and theranostic applications. From this point of view, Au P-GERTs are the optimal candidates for future diagnostic and therapeutic applications owing to their simultaneous extra-high SERS response, ultra-photostability, and multiplexing capability 39. On the other hand, the P-GERTs have a slightly larger particle size that is beyond the typical limit for kidney clearance *in vivo*, which raises the biosafety issue. One could expect clinical trials of such labels only after their toxicity and clearance efficiency are studied. Nevertheless, a very recent report of a clinical trial with similarly structured Au-silica nanoshells (150 nm in average size) for the focal ablating of prostate tumors showed that these particles are safe for use, yielding promising initial results 115. In addition, some protocols are available to synthesize Au@RM@Au-Ag GERTs with a size as small as 25 nm and with relatively low polуdispersity 61-63.

One possible application for GERTs is as the second near-infrared window (NIR-II) SERS tags. In recent years, biomedical imaging in the NIR-II region of 1000-1700 nm has garnered much attention because of its enormous improvement in the imaging depth and spatial resolution by reducing the scattering, absorption, and autofluorescence of biological tissues in this region 116. So far, a variety of nanomaterials have already been developed for use as NIR-II probes, such as carbon nanotubes, quantum dots, and lanthanide NPs 117-119. To the best of our knowledge, the studies and applications of SERS imaging in the NIR-II region are still underdeveloped owing to the lack of suitable NIR-II SERS tags. The GERTs, however, hold promise as NIR-II probes because of their core-shell structure: by controlling the gap size to tune the plasmonic coupling between the core and the shell, it is possible to adjust the longer-wavelength mode (i.e., lower energy resonance) to the second window, which leads to prominent EM enhancement in this region 120. Further work is needed for this demonstration.

We have witnessed successful biomedical applications of GERTs from biodetection to theranostics, although most of these applications are currently in the initial stage. The performance of GERTs as optical labels in biodetection could be improved by the advances in instruments. Because of the outstanding sensitivity of GERTs and their multiplex capability owing to the inner nanogap, microfluidic chips and SERS flow cytometry for high-throughput detection are needed in future clinical analysis 121-123. Particularly, SERS-image guided surgery holds great promise in clinical applications, considering that a Raman intraoperative device (Raman-pen) was used in trials. So far, enormous advancements have been obtained in the Raman-guided surgery with GERTs on several tumor types. Together with the development of brighter GERTs, future studies in this field could also be focused on the improvement of the specificity and imaging speed. The specificity of GERTs toward tumors is critical for the precise imaging and resection. Also, the interaction of GERTs with biological environments should be systematically studied and evaluated for *in vivo* applications; and different biofunctionalization strategies should be developed accordingly. Precision could be improved by using better methodologies of imaging, among which the dual-tracer strategy could be considered. This has been widely applied in fluorescence imaging for decades 124. Typically, a specific and a nonspecific isotype of biofunctionalized GERTs could be used, and the location or the margin of the tumors could be obtained through the subtraction between targeted and untargeted tags. As for the imaging speed, Raman systems are developing rapidly 125, as are commercial spectrometers. But the fastest imaging speed in the current literature (6 s for a single cell) is still too low for practical use on humans, lagging far behind that of fluorescence imaging. Continuous efforts are needed to improve imaging speed. Moreover, GERTs are ideal tools for multimodal detection and imaging, as well as for combined photothermal- and chemo-therapy because of their plasmonic effects and high loading capacity for drugs and additional imaging agents owing to the core-shell structure. This provides better effects in the diagnosis and treatment of microtumors.

To summarize, most of the current studies have attempted to demonstrate the proof of concept of their chemistry and related optical properties, and we believe that these tags are still underestimated and should be more extensively tested in biomedical applications, especially toward intraoperative navigation in clinical translation. With account taken of their tremendous promise and superior characteristics over conventional SERS tags, GERTs will surely open new possibilities in Raman technology.

## Figures and Tables

**Table 1:** Reported SERS EFs for different GERTs.

Refs	SERS tag structure	Raman reporter	Laser wavelength, nm	Measured EF
31	Au core 20 nm@gap 1 nm@Au semishell	Cy3	660, 785	2×10^8^
	Au core 20 nm@gap 1 nm@Au shell	Cy3	532	4×10^8^
32	Au core 15 nm@gap 1.1 nm@Au shell 25 nm	4,4'-dipyridyl and 5,5'-dithiobis(2-nitrobenzoic acid)	633	1×10^8^
5	Au core 12 nm@gap 1 nm@ Au shell	ROX, Cy3, 4,4'-dipyridyl	633	1.1×10^8^ -2.6×10^9^
48	Au core 12 nm@gap 1 nm@ Au shell	Cy3, Cy5, TAMRA	514, 633, 785	1×10^6^-1×10^8^
	AuNR core @gap 1 nm@ Au shell	Cy3, Cy5, TAMRA	514, 633, 785	1×10^8^
27	Au core 12 nm@gap 1 nm@ Au shell	4,4'-dipyridyl	633	1.3×10^8^
74	Au 20 nm@reporter@Ag shell	rhodamine B	785	2.3×10^5^-5×10^5^
45	Au 20 nm@reporter@Ag shell	toluidine blue	785	3×10^5^
35	AuNRs@reporter@Ag shell	4-aminothiophenol	785	6.5×10^6^-4×10^7^
56	Au 20 nm@reporter@Ag shell	mercaptobenzoic acid	785	2.3×10^5^
75	AuNSt@reporter@Ag shell	mercaptobenzoic acid	785	4.2×10^6^
18	Au core 20 nm@gap 0.62 nm@Au shell	1,4-benzenedithiol	785	1.7×10^11^
19	Au core 20 nm@gap 1.5 nm@Au shell	rhodamine B	785	1.4×10^8^
	Au core 20 nm@gap 5 nm@Au shell			1.1×10^7^
	Au core 20 nm@gap 11 nm@Au shell			2.7×10^6^
19	Au core 20 nm@gap 2 nm@Au shell	rhodamine B	785	8.8×10^7^
	Au core 20 nm@gap 7 nm@Au shell			2×10^7^
	Au core 20 nm@gap 13 nm@Au shell			9.6×10^6^

**Table 2:** Comparison of GERT-based detection of biomarkers.

Refs	Assay substrate	GERT structure	Raman reporter	Targeting ligand	Biomarker	LOD
92	96-well plate	Au nanosphere core, Ag shell	crystal violet	biotinylated antibody	PSA	19.2 pM
			rhodamine B		CRP	7.7 pM
46	nitrocellulose membrane strip	Au nanorod core, Au shell	4-NBT	anti-cTnI monoclonal antibodies	cTnl	0.1 ng/mL
60	nitrocellulose membrane strip	Au nanosphere core, Au-Ag double-shell	4-NBT	HCG antibody	HCG	0.025 mIU
51	magnetic bead	Au nanostar core, Au shell	1,4-BDT	anti-IgG polyclonal antibody	mouse IgG	0.1 pg/mL
62	magnetic bead	Au nanosphere core, Au-Ag shell	4-mercaptopyridine	complementary DNA	HAV DNA	10-100 aM
98	nitrocellulose membrane	Au nanosphere core, Au shell	4-NBT	anti-rabbit antibody	rabbit IgG	2.3 µg/mL
			2-Naphthalenethiol	anti-human antibody	human IgG	4.7 µg/mL
			4-acetamidothiophenol	anti-chicken antibody	chicken IgG	9.4 µg/mL
27	*magnetic bead	Au nanosphere core, Au shell	4,4'-dipyridyl	complementary DNA	HAV DNA	0.39 pM
			5,5'-dithiobis(2-nitrobenzoic acid)	complementary DNA	hepatitis B virus (HBV) DNA	0.18 pM
			phthalazine	complementary DNA	human immunodeficiency virus (HIV) DNA	0.51 pM

*Simultaneous multiplex analysis of three types of DNA.

## Abbreviations

ATP: 4-aminothiophenol
AuNR: Au nanorod
Au NP: Au nanoparticle
AuNS: Au nanostar
BDT: 1,4-benzenedithiol
CRP: c-reactive protein
CTAC: cetyltrimethylammonium chloride
cTnI: cardiac troponin I
DDA: discrete dipole approximation
EM: electromagnetic
EF: enhancement factor
FDTD: finite difference time domain
GERTs: gap-enhanced Raman tags
HAV: hepatitis A virus
HBT: 4-hydroxybenzenethiol
HBV: hepatitis B virus
HCG: human chorionic gonadotropin
HRTEM: high resolution transmission electron microscopy
IgG: Immunoglobulin G
LFIA: lateral flow immunoassay
LOD: limit of detection
MBA: mercaptobenzene acid
MBT: methylbenzenethiol
MPBA: mercaptophenylboronic acid
NBT: nitrobenzenethiol
NIR: near-infrared
NM: nanomatryoshka
NP: nanoparticle
PDA: polydopamine
P-GERT: petal-like GERT
PR: plasmon resonance
PSA: prostate-specific antigen
PTT: photothermal therapy
RM: Raman molecule
SERS: surface-enhanced Raman scattering (spectroscopy)
S-GERT: smooth GERT
TEM: transmission electron microscopy
